# Evaluation of the Genetic Diversity and Differentiation of Black Locust (*Robinia pseudoacacia* L.) Based on Genomic and Expressed Sequence Tag-Simple Sequence Repeats

**DOI:** 10.3390/ijms19092492

**Published:** 2018-08-23

**Authors:** Qi Guo, Xiuyu Li, Shuhong Yang, Zhiheng Yang, Yuhan Sun, Jiangtao Zhang, Sen Cao, Li Dong, Saleem Uddin, Yun Li

**Affiliations:** 1 Beijing Advanced Innovation Center for Tree Breeding by Molecular Design, National Engineering Laboratory for Tree Breeding, Key Laboratory of Genetics and Breeding in Forest Trees and Ornamental Plants, College of Biological Sciences and Technology, Beijing Forestry University, Beijing 100083, China; Guoqi0529@126.com (Q.G.); 18730281237@163.com (X.L.); sunyuhan@bjfu.edu.cn (Y.S.); sailingcs@163.com (S.C.); dongli0408@126.com (L.D.); saleemkhan86@hotmail.com (S.U.); 2 Henan Academy of Forestry, Zhengzhou 450008, China; yangshh0315@163.com (S.Y.); hnlky@163.com (J.Z.); 3 State-Owned Linghai Hongqi Forest, Jinzhou 121228, China; yzh13591256494@163.com

**Keywords:** EST-SSR, genetic differentiation, genetic diversity, genomic-SSRs, *Robinia pseudoacacia* L.

## Abstract

Understanding the genetic diversity and differentiation of the genetic resources of a species is important for the effective use and protection of forest tree resources. Ex situ development is a common method for the protection of genetic diversity and an essential resource for users who require ready access to a species’ germplasm. In this study, we collected seeds of black locust (*Robinia pseudoacacia* L.) from 19 provenances, covering most of its natural distribution; we randomly selected 367 tender leaves with well-grown and different maternal strains from this group for further analysis. Forty-eight simple sequence repeat (SSR) primers were successfully selected from 91 pairs of SSR primers using native-deformation polyacrylamide gel electrophoresis. In addition, we identified identical genotypes among all individuals and evaluated the quality of the markers. From this, 35 loci were confirmed for analyses of genetic diversity and differentiation of the black locust provenances, which contained 28 expressed sequence tag-derived simple sequence repeats (EST-SSRs) and 7 genomic DNA-derived simple sequence repeats (G-SSRs). We observed high genetic diversity among the native black locust provenances, from which Wright’s fixation index and molecular variance suggested that a majority of the genetic differentiation variation could be attributed to within-provenance differences. The genetic distance and identity results indicated that geographic distance was not a dominating factor influencing the distribution of black locust. This is the first study to evaluate provenance genetic variation in native black locust samples using two types of SSR markers, which provides a comprehensive theoretical basis for ex situ conservation and utilization of genetic resources, with an emphasis on breeding applications.

## 1. Introduction

Genetic variation can reflect the adaptation potential of species driven by long-term evolution via mutations, genetic drift, gene flow, and natural selection, which support rich genetic variation within species [1,2,3]. During long-term introduction, hybridization, and breeding of many plant species, populations increase and become widely distributed [4]. However, forest tree breeding is time-consuming and labor-intensive, with slow progress due to the general characteristics of trees, such as their perennial life cycle, height, and high heterozygosity. A comprehensive understanding of the genetic diversity and differentiation of genetic resources is important, as a reduction of genetic variation may compromise the plasticity and the resilience of a species in response to biotic and abiotic stresses [5]. The identification, selection, and preservation the genetic of diversity within forest trees species is therefore of particular interest due to the long life spans of these organisms, which require adaption to different environments and climate. These characteristics can be successfully used to inform conservation, efforts, including targeted breeding programs seeking to improve genetic diversity within a provenance [5,6,7,8].

In situ and ex situ conservation are two common methods for protecting of plant genetic resources and the latter serves as an essential genetic resource for users who require ready access, to a species’ germplasm. Ex situ is defined as the conservation of plants samples/seeds away from their field habitats with the goal of genetic improvement and conservation as a means of combating the genetic erosion (loss of useful genes) due to natural and human-mediated disorders [9,10,11]. Black locust (*Robinia pseudoacacia* L.) is a nitrogen-fixing tree native to the Appalachian and Ozark Mountains of the eastern United States. It is a widespread deciduous broadleaved tree with excellent characteristics of poor soil tolerance and resistance to salinity and alkalinity and with substantial ecological and environmental value [12]. However, the genetic variation of black locust in the United States has not been studied, likely due to limitation brought about by the production of fence posts in the Appalachians, as well as the stability of mines and roads in these regions [13]. Black locust has been introduced in many countries worldwide for the purpose of ex situ conservation as well as for its adaptability, rapid growth, and high bioenergy potential [12,14,15,16]. For example, it has become an important ecological afforestation tree species in China [17,18,19].

Unfortunately, there are few investigations of the genetic diversity and differentiation of black locust seeds collected within its natural distribution, and all ex situ conservation analyses of its genetic diversity performed to date have been based on allozymes [13,20,21], amplified fragment length polymorphisms [22], and inter-simple sequence repeats [23]. Among the various molecular marker technologies developed to date, simple sequence repeat (SSR) markers represent an ideal option for plant-based genetic diversity analyses, as they are codominant, hypervariable, amplification-stable, highly informative, inexpensive, and highly efficient [24,25,26]. Previous studies examining the genetic diversity of black locust using microsatellite markers have been extremely limited in scope, Lian et al. (2002, 2004) [27,28] and Mishima et al. (2008) [29] isolated 7, 3, and 11 polymorphic microsatellite loci, respectively, to assess the diversity of genotypes of black locust. Liesebach et al. (2012) [30] discovered a weak linkage between some microsatellite loci, although the methodology used for fingerprinting and parentage analysis remains questionable. In a more extensive analysis, Malvolti et al. (2015) [31] reported the diversity or identity of 91 black locust clones by root cuttings, although diversity analysis was performed using only nine genomic DNA-derived SSR (G-SSR). Although these studies constitute an excellent start for assessing the genetic diversity of the black locust germplasm, such efforts are obviously insufficient due to the long-term dynamic nature of natural and biological adaptability [32]. Additionally, the selection of suitable SSR markers remains an essential step when studying genetic differences in germplasm resources, which was not fully established in these studies. SSR markers include genomic DNA-derived SSRs (G-SSRs) and expressed sequence tag-derived SSRs (EST-SSRs). EST-SSRs were derived from expressed regions of the plant genome, showing a higher range of cross-transferability among several related genera/species, while G-SSRs display greater polymorphism within and between species. As these markers are derived from expressed regions of the plant genome, they provide greater insight into the conservation of a given region [33,34,35]. For example, in human neurons, an intronic polymorphic TCAT repeat in the tyrosine hydroxylase (*TH*) gene may regulate transcription via the microsatellite HUMTH01 [36]. Accordingly, by using two complementary marker types, each with its respective advantages, we are able to better asses the genomic differences within and between the species.

In this study, we evaluated the genetic variation of black locust provenance, based on G-SSR and EST-SSR markers. We used seeds collected from the distribution of black locust in the United States as well as ex situ conserved samples from Henan province, China. The aims of our study were to clarify the genetic diversity among and within provenances, the genetic differentiation among provenances, the degree of provenance genetic differentiation, and the correlation between the genetic and geographic distance of the analyzed provenances to provide a theoretical basis for improved evaluation, use, and protection of black locust genetic resources.

## 2. Results

### 2.1. Polymorphism and Determination of SSR Markers

We screened 91 pairs of SSR primers using polyacrylamide gel electrophoresis (PAGE) in eight black locust samples from different sources. Of these, 43 pairs failed to amplify clear PCR products, while 48 exhibited successful amplification. The rate of polymorphism was 52.75%. This set of polymorphic SSR primers included 37 EST-SSRs and 11 G-SSRs (Table S1).

The set of 48 primer pairs was further analyzed to obtain suitable SSR markers for subsequent analyses of the black locust provenances. Four of the markers (Rp-11, Rply1, Rply32, and Rops16) had more than 20% missing locus data. Genotype pairing was performed on all individuals; no two samples had an identical genotype. The Ewen–Watterson test was performed, four loci (Rops02, Rops15, Rops16, and Rp200) with significant deviations from a neutral equilibrium model. In addition, Micro-Checker analysis detected eight loci (Rp-08, Rp-19, Rp-30, Rp-40, Rply1, Rply3, Rply32, and Rply44) possessing null alleles. After combining these results, 13 pairs of SSR markers were ultimately deleted, and the remaining 35 markers, consisting of 28 EST-SSRs and 7 G-SSRs, were used for further analyses.

### 2.2. Characteristics of EST-SSRs and G-SSRs

An analysis of nucleotide sequences generated using polymorphic primers for EST-SSRs and G-SSRs showed that, in *Robinia pseudoacacia* L., the EST-SSRs consisted of 35.714% trinucleotide repeats, 28.571% hexa-nucleotides repeats, 17.857% penta-nucleotide repeats, 10.714% tetra-nucleotide repeats and 7.143% di-nucleotide repeats. In contrast, the G-SSRs were composed of only di-nucleotide repeats (Table 1). However, the number of G-SSR alleles was almost double that of EST-SSRs, yielding 1.62 times the level of polymorphism as that of EST-SSR (Table 2). Nevertheless, the amplification efficiency of EST-SSRs was as high as 65.079% after PCR amplification (Table 3).

### 2.3. Genetic Diversity Analysis

**Figure 1:**
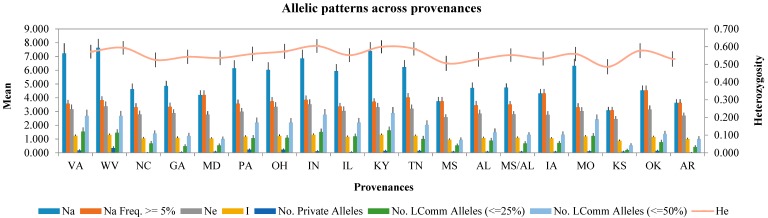
Average genetic diversity indices for the 19 provenances of *Robinia pseudoacacia* L. analyzed in this study. *Na*: No. of Different Alleles; Na Freq. ≥ 5%: No. of different alleles with a frequency ≥ 5%; *Ne*: No. of effective alleles = 1/(Sum pi^2^); *I*: Shannon’s Information Index, *I* = −1 Sum (pi Ln (pi)); No. Private Alleles: No. of alleles unique to a single provenance; No. LComm Alleles (≤25%): No. of locally common alleles (Freq. ≥ 5%) found in 25% or fewer provenances; No. Lcomm Alleles (≤50%): No. of locally common alleles (Freq. ≥ 5%) Found in 50% or fewer provenances; *He*: Expected heterozygosity, *He* = 1 − Sum pi^2^. The error line in each column is the standard error values of corresponding diversity index.

We used the 35 SSR markers to evaluate genetic diversity at the locus and provenance levels. At the locus level, 439 alleles were identified, and the number of alleles per locus (*N*) ranged from 3 (at Rp-21) to 34 (Rops18) (Table S2). Meanwhile, the number of effective alleles per marker ranged from 1.065 (Rp-44) to 6.552 (Rops18) (mean: 2.988). The variations in polymorphism information content (*PIC*) and gene diversity (*H*) were consistent, with the highest values at Rops18 (0.898 and 0.906, respectively), followed by Rops05 (0.886 and 0.895), and the lowest at Rp-44 (0.061 and 0.061) (mean: 0.566 and 0.595). The expected heterozygosity (*He*) ranged from 0.054 (Rp-44) to 0.837 (Rops05) (mean: 0.553), while the observed heterozygosity (*Ho*) ranged from 0.044 (Rp-44) to 0.835 (Rp-38) (mean: 0.495). At the provenance level, the genetic diversity index was used to assess the variance of heterozygosity (Figure 1 and Table S3), with the highest values (No. of effective alleles (*Ne*) = 3.527) observed for the IN provenance and the lowest values (*Ne* = 2.457) for the KS provenance. In terms of uniformity, Shannon Information index values (*I*) ranged from 0.867 (IN provenance) to 1.306 (KS provenance). The IN and KY provenances showed the highest levels of expected heterozygosity (0.604 and 0.599, respectively). In total, 81 private alleles (PAs) from all 19 provenances were identified in this study (average frequency = 0.343), with the highest number (12 PAs) found in the WV provenance (Table S4) The No. of Different Alleles (*Na*) estimated for each provenance ranged from 3.086 (KS) and 7.629 (WV). Furthermore, Wright’s fixation index (*F_ST_*) values among global and pairwise multi-loci ranged from 0.040 (Rp-12) to 0.110 (Rops08) and 0.007 (WV vs. Kentucky) to 0.089 (Kansas vs. Arkansas), respectively (Tables S2 and S5).

### 2.4. Provenance Differentiation Analysis

Analysis of molecular variance (AMOVA) was performed for all black locust accessions from the 19 study areas to analyze the genetic variation between and within provenances. The variance accounted for 3% of the variance among the provenances and 97% within provenances (Table 4).

**Figure 2:**
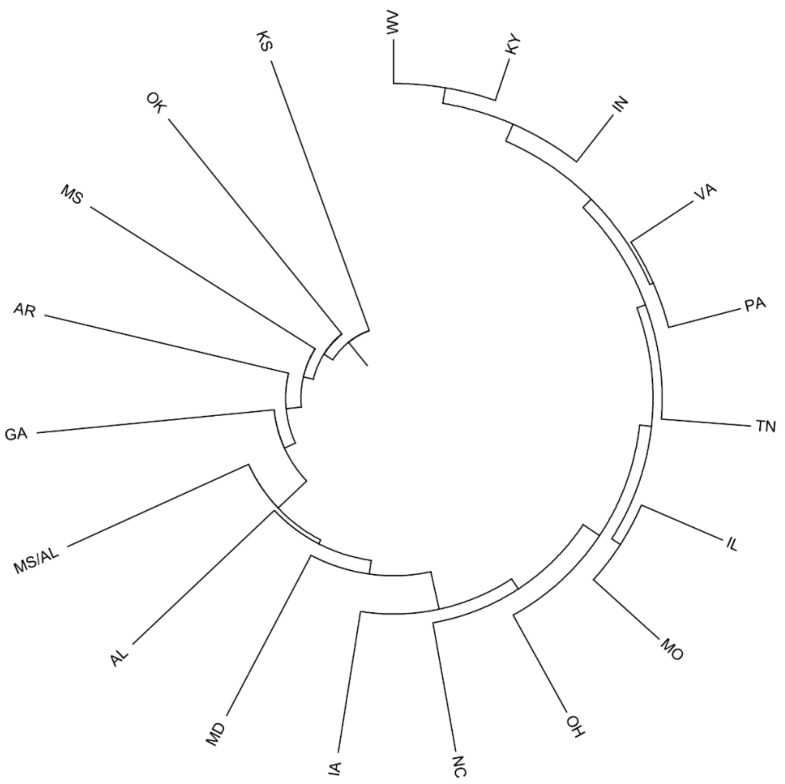
UPGMA dendrogram based on Nei’s (1972) genetic distance values showing the relationship among different provenances of *Robinia pseudoacacia* L.

**Figure 3:**
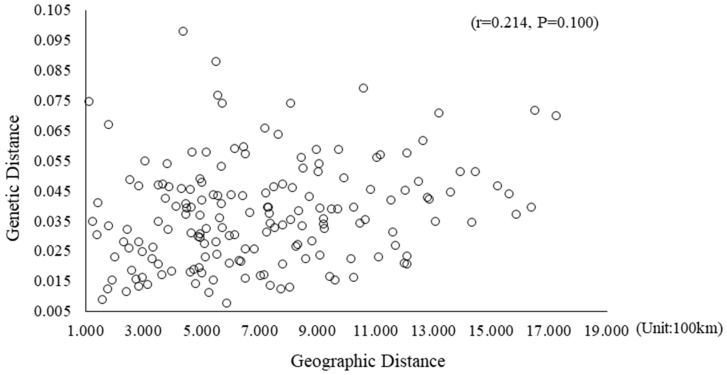
Relationship between genetic distance and geographic distance for all black locust provenances (The *X*-axis scale is 0.010, and the *Y*-axis is 2.000). Genetic distance was computed based on pairwise *F_ST_*/(1 − *F_ST_*) estimates among provenances.

Nei’s unbiased genetic distance and identity measure revealed genetic distance and identity values ranging from 0.002 to 0.141 and from 0.869 to 0.998, respectively, between provenances (Table S6). Among all area pairs, the WV and KY provenances showed the closest genetic distance (0.002) and highest identity (0.998). Using Nei’s genetic distance values, an UPGMA (Unweighted Pair Group Method with Arithmetic Mean) dendrogram was constructed showing the phylogenetic relationship among the 19 provenances (Figure 2). The geographic distance matrices ranged from 998 to 17,271 km based on latitude and longitude values computed using GenAlEx ver. 6.501 (details not shown). MD and OK represented the farthest geographic distance (17,271 km) but not the farthest genetic distance. Meanwhile, OK and KS represented the closest geographic distance (998 km) but not the closest genetic distance. Mantel tests (Figure 3) among the provenances and geographic distance showed consistent results.

## 3. Discussion

The microsatellite markers used in this study consisted of both expressed sequence tag-derived SSRs (EST-SSRs) and genomic DNA-derived SSRs (G-SSRs). A total of 37 EST-SSRs and 11 G-SSRs were confirmed as polymorphic based on results from a 6% native-deformation PAGE for eight plant materials with different phenotypes and sources. In this study, the levels of polymorphisms and allele numbers were higher for G-SSRs compared to EST-SSRs, with mean *PIC* values of 0.817 and 0.503, respectively. Diversity analyses found G-SSRs to be more polymorphic than EST-SSRs. These results are consistent with those of other studies, including those on both barley (mean *PIC* = 0.727 for 48 G-SSRs and 0.581 for 16 EST-SSRs) [37] and *Linum usitatissimum* L. (mean *PIC* = 0.326 of 15 G-SSRs and 0.245 for 7 EST-SSRs) [38]. Nevertheless, the amplification efficiency of EST-SSRs was 65.079%, showing higher conservation than that of G-SSRs (52.991%) in nine related species (*Gleditsia sinensis*, *Cercis chinensis*, *Wisteria sinensis (Sims) Sweet*, *Trifolium repens*, *Amorpha fruticose*, *Sophora japonica*, *Sophora japonica var. pendula, Robinia pseudoacacia* ‘Frisia’ and *Robinia pseu-doacacia* var. *decaisneana*). The high rate of EST-SSRs amplification in related species may be derived from a conserved region of the plant genome, which is consistent with the lower mutation frequency seen for ESTs compared to that of genomic DNA [39,40]. A recent study of human neurological tissues found that different microsatellite repeats could have subtle effects on the regulation gene transcription [36]. The analysis of the nucleotide sequences described here using two different SSR markers found that EST-SSR primers produced a range of mono- to hexa-nucleotides DNA segment repeats, compared with G-SSRs, which produced only tetra-nucleotide repeats. In short, EST-SSRs and G-SSRs have their own advantages for genomic studies assessing both inter- and intra- species diversity, with the complementary nature of this two-marker system serving as an ideal method for the study of *Robinia pseudoacacia* L.

Simple sequence repeats have emerged as one of the most popular and versatile marker types for plant genetics [34,35,36]; however, insufficient details regarding the practicalities of different genetic tools and a lack of validation based on blind analyses of existing data sets may pose a risk for researchers when judging their own results [41]. Here, we evaluated a set of 48 pairs of polymorphic primers, expecting to improve the efficiency of the study. From this set of 48 primer pairs, four non-neutral loci were removed by a neutral equilibrium model, suggesting that these loci were possibly affected by selection or other factors [42]. Meanwhile, eight loci had null alleles and likely contributed to the positive inbreeding coefficient [43]. Ultimately, 35 primer pairs were identified for further analysis.

Microsatellite markers are an essential tool for gene discovery, enabling the detection of distinctive alleles that can be useful for studying marker-trait associations in populations [44]. In this study, we found an average of 12.594 observed alleles per locus within the 367 black locust genotypes. This value was higher than the level of *N* (3.2) in seedings obtained from seed collections representing the natural range of black locust that was determined using the allozyme method [13], and it was higher than the mean value (*N* = 7.429) seen in 18 *R. pseudocacia* L. individuals, which was computed based on seven G-SSR markers [27]. The high number of alleles observed in his study may be due to the relatively large sample size used in this study in addition to the use of two types of SSR markers. In terms of diversity, the mean Shannon’s Information index value (*I*) was 1.133 across all 19 provenances, substantially higher than that seen in *R. pseudocacia* L. samples collected from the 10 main planting districts in China, as determined based on 10 pairs of AFLP (*I* = 0.138) and ISSR markers (*I* = 0.373) [22,23]. This considerable difference in Shannon’s Information index values may be related to the different sources of materials and/or the different types of molecular markers used.

Observed and expected heterozygosity are two important parameters for gauging gene diversity [45]. In this study, the mean observed (*Ho*) and expected (*He*) levels of heterozygosity were 0.495 and 0.553, respectively. These levels are somewhat lower than those seen in two previous reports of black locust diversity performed using G-SRR markers. Lian et al. (2002) [27] reported *Ho* and *He* levels of 0.615 and 0.773; 7 G-SSR markers, respectively, compared with 0.661 and 0.739, respectively, in Mishima et al. (2008) [29]. Low levels of heterozygous individuals were also observed within the provenances (Table S5). The mean value of *Ho* was lower than that of *He*, at both the locus and provenance levels, indicative of a deficit of heterozygotes in black locust. In addition, *F_IS_* was 0.105 and 0.133 at the locus and provenance levels, respectively, possibly related to a degree of self-intersection in this species. A similar result was reported in a study of black locust allozymes (*F_IS_* = 0.072) [13]. These results suggest a low level of heterozygous deletions that are due either to the spatial scale chosen for sampling, in which the study site was larger than the true scale of a population, or to inbreeding, which might be attributable to the hybridization of only a few related species within the natural distribution over a long period [41,46].

The degree of genetic differentiation in provenances based on *F_ST_* is usually defined as low (*F_ST_* < 0.05), medium (0.05 > *F_ST_* > 0.15), or high (*F_ST_* > 0.15) [34]. Based on these criteria, the provenances exhibited a low degree of differentiation. This was confirmed by AMOVA, which indicated that only 3% of genetic variation appeared among provenances, the majority of which was attributed to within-provenance differences. Such within-provenance variation might be a characteristic of woody plants with long growth periods and outcrossing, resulting in increased genetic diversity among individuals within provenance and reduced genetic differentiation among provenances [35]. Additionally, the values of Nei’s unbiased genetic distance (D) and identity (I) also showed the genetic differentiation between provenances. The UPGMA dendrogram, constructed using Nei’s algorithm (1972) and other indices of genetic variability, showed low values of genetic distance among the 19 analyzed provenances. Mantel tests between provenance and geographic distance showed consistent results. These findings provide further evidence that geographic distance is not a dominant factor influencing the distribution of black locust. This was similar to the results of Surles et al. [13], who reported a lack of significant correlation between genetic identity and geographic distance based on an allozyme study. Taken together, these results all indicate that the primary variation comes from individuals of black locust, rather than from the genetic and geographic distance between provenances. It provides a theoretical basis for the selective breeding of *Robinia pseudoacacia* L., and shows that black locust may possess favourable adaptation due to the abundant variation among individuals in a provenances.

## 4. Materials and Methods

### 4.1. Plant Materials

Open-pollinated *R. pseudoacacia* fruits were collected from the 19 main black locust distribution areas in the United States (including 18 states and 1 state border) from September to October 2010, which includes most of the natural distribution of black locusts (Table 5) [12,13,14]. In these regions, several fruits of black locusts with normal growth and a diameter at breast height greater than 20 cm were collected in 500-m intervals, and the obtained fruits were mailed to the Henan Academy of Forestry Sciences in China. From April to July 2011, the collected seeds were soaked for 24 h in 20 areas. After the seedlings were successfully germinated and approximately 30 cm tall, they were transplanted to two test sites in Mengjin (34°49′18′′ N, 112°28′12′′ E) and Minquan (34°43′59′′ N, 115°05′10′′ E) in Henan and scientifically managed to preserve normal seedling growth. Three hundred and sixty-seven black locust tender leaves with well-grown and different maternal strains were randomly selected from the above two test bases as experimental material in 2017. Information on the experimental samples is presented in Table S7. Tender leaves were randomly collected from trees and rapidly placed in sealed bags containing silica gel. Then, the plant materials were transferred to the National Engineering Laboratory for Tree Breeding, Beijing Forestry University, China (40°0′22′′ N, 116°21′1′′ E) and protected from light at room temperature until DNA extraction

### 4.2. DNA Extraction

For every sample, total genomic DNA was isolated from dried and tender leaves using a plant genomic DNA kit (Tiangen, Beijing, China). After extraction, to check the quality and concentration, 1 µL genomic DNA from each genotype was loaded onto a 1% agarose gel and quantitated using the NanoDrop 2000 (Thermo Fisher Scientific, Wilmington, DE, USA). All DNA samples were normalized to an equal concentration (20 ng/µL) using TB elution buffer (Tiangen) and stored immediately at −80 °C for SSR amplification.

### 4.3. SSR Marker Screening

We assessed 91 pairs of SSR primers, including 21 pairs of G-SSR primers found in the literature [27,28,29] and 60 EST-SSRs (Table 6) [47,48]. Details of the SSR primers which were synthesized by TSINGKE Biological Technology Co., Ltd. (Beijing, China) are reported in Table S8.

To obtain SSR primer sets with rich polymorphism and steady bands for subsequent experiments, we selected eight plant materials with different phenotypes and sources that had been investigated by black locust breeders in China using a screening assay. Table S9 presents information on the samples. Eight samples were collected from two artificial planting distribution regions in China: Daqingshan Forest Farm, Fei Country, Linyi, Shandong Province (35°23′ N, 118°11′ E) and Mengjin, Luoyang, Henan Province (34°49′ N, 112°28′ E). PCR amplification was performed in a total reaction volume of 20 µL containing 10 µL 2× TSINGKE^®^ Master Mix (blue) (TSINGKE Biological Technology Co., Ltd.), 1 µL of the forward primer (10 µM), 1 µL of the reverse primer (10 µM), 2 µL genomic DNA (20 ng/µL), and 6 µL double-distilled water. PCR amplification was performed by an initial denaturation step at 94 °C for 4 min, followed by 20 cycles of 94 °C for 30 s, 60 °C to 50 °C for 30 s (decreasing by 0.5 °C per cycle), and 72 °C for 1 min, 20 cycles with the annealing temperature fixed at 50 °C, 72 °C for 45 s, and a final extension at 72 °C for 10 min using the BIO-RAD T100 thermal cycler [49]. The amplified PCR products from the SSR primer screening were resolved by 6% native-deformation PAGE using 1× Tris–borate–EDTA buffer solution at 220 V for 100 min. After electrophoresis, the amplified products were rinsed, silver-stained, visualized under ultraviolet light, and photographed.

### 4.4. Comparison of EST-SSR and G-SSR outcomes

The effectiveness comparison of EST-SSR and G-SSR amplification in several genera of Leguminosae, including *Gleditsia* (Caesalpinioideae), *Cercis* (Caesalpinioideae), *Wisteria* (Papilionoideae), *Trifolium* (Papilionoideae), *Amorpha* (Papilionoideae), *Sophora* (Papilionoideae), and *Robinia* (Papilionoideae), was used to evaluate SSR markers. Markers were obtained from Beijing Forestry University Campus, China (40°0′22′′ N, 116°21′1′′ E) [48]. Genomic DNA extraction, PCR amplification, and fluorescence modification were performed as described above.

### 4.5. SSR Amplification of Provenances

Based on the screening, optimal primers were applied to the provenance of 367 samples for amplification analysis. SSR amplification was performed by PCR in a 20-µL reaction volume containing 10 µL 2× TSINGKE^®^ Master Mix (blue) (TSINGKE Biological Technology Co., Ltd.), 0.1 µL forward primer (10 µM), which was labeled at the 5′ end with fluorescent-dye (FAM, HEX, ROX, and TAMRA), 0.3 µL reverse primer (10 µM), 0.2 µL M13 primer (10 µM; 5′-TGTAAAACGACGGCCAGT-3′), 2 µL genomic DNA (20 ng/µL), and 7.9 µL sterile double-distilled water [42]. The PCR amplification conditions were as follows: 4 min at 94 °C, 20 cycles of 30 s at 94 °C, 30 s at 60 °C to 50 °C (decreasing at 0.5 °C per cycle), and 1 min at 72 °C, 20 cycles of 30 s at 94 °C, 30 s at 50 °C, and 45 s at 72 °C, and a final extension at 72 °C for 10 min.

The PCR products were separated using the ABI 3730XL DNA capillary electrophoresis analyzer (Applied Biosystems, Foster City, CA, USA) and a GeneScan-500LIZ size standard. Alleles of the SSRs were confirmed using GeneMarker ver. 2.2.0 (SoftGenetics LLC, State College, PA, USA).

### 4.6. Genetic Diversity and Differentiation Analysis

The *N*, *Na*, *Ho*, *He*, percentage of polymorphic loci, and Shannon’s information index were computed using GenAlEx ver. 6.501 [50,51]. Calculations of Wright’s F-statistics (*F_IS_*, *F_IT_*, and *F_ST_*) among provenances were estimated using FSTAT ver. 2.9.3 software [52]. *PIC* values were calculated using PowerMarker ver. 3.25 [53]. Hardy–Weinberg equilibrium was calculated using Arlequin ver. 3.5 [54] with 100,000,000 steps in the Markov chain and 100,000 dememorization steps [55], and the Ewen–Watterson test was used to detect whether the polymorphic microsatellite markers deviated significantly from neutral equilibrium [56]. AMOVA was performed using GenAlEx 6.5 to estimate the genetic variance among and within provenances and geographic areas. Simultaneously, Nei’s genetic distance was estimated using GenAlEx 6.5. Null alleles were detected using Micro-Checker ver. 2.2.3 [57]. Nei’s genetic distance values were used to construct the UPGAM dendrogram comparing genetic diversity across different provenances using PowerMarker ver. 3.25. Finally, GENECAP ver. 1.4 was used to identify materials with identical genotypes [58].

## 5. Conclusions

The present study revealed that (1) the genetic diversity among the native black locust populations is high, (2) the majority of genetic differentiation variation was attributed to differences within populations, and (3) geographic distance is not a dominant factor influencing the distribution of black locust. These findings provide comprehensive information that can be used as a foundation for genetic assessments, improvements, and management of black locust.

## Figures and Tables

**Table 1:** Characteristics of expressed sequence tag-derived SSRs (EST-SSRs) and genomic DNA-derived SSRs (G-SSRs) markers in *Robinia pseudoacacia* L. individuals.

Repeat Type	EST-SSR Polymorphic Number	Remaining Number after Screening	Percentage	G-SSR Polymorphic Number	Remaining Number after Screening	Percentage
Di-	4	2	7.143	11	7	100
Tri-	14	10	35.714	-	0	0
Tetra-	3	3	10.714	-	0	0
Penta-	7	5	17.857	-	0	0
Hexa-	9	8	28.571	-	0	0
Total	37	28	100	11	7	100

“-” means no polymorphic.

**Table 2:** Diversity of EST-SSRs and G-SSRs in *Robinia pseudoacacia* L.

SSR Marker Type	Genotype Number	Allele Number (*N*)	Gene Diversity (*H*)	Polymorphism Information Content (*PIC*)
EST-SSR	24.286	10.464	0.535	0.503
G-SSR	65.429	20.857	0.835	0.817

**Table 3:** Amplification efficiency in Leguminosae species using EST-SSRs and G-SSRs.

Species	EST-SSR Number of Loci	Percentage	G-SSR Noumber of Loci	Percentage
*Gleditsia sinensis*	20	57.143	8	61.538
*Cercis chinensis*	20	57.143	6	46.154
*Wisteria sinensis* (Sims) *Sweet*	21	60.000	8	61.538
*Trifolium repens*	17	48.571	0	0.000
*Amorpha fruticosa*	19	54.286	8	61.538
*Sophora japonica*	21	60.000	9	69.231
*Sophora japonica* var. *pendula*	21	60.000	7	53.846
*Robinia pseudoacacia* ‘Frisia’	34	97.143	7	53.846
*Robinia pseu-doacacia* var. *decaisneana*	32	91.429	9	69.231
Mean	22.778	65.079	6.889	52.99

**Table 4:** Analysis of molecular variance (AMOVA) of the 19 black locust provenances based on SSR markers.

Source of Variation	Degree of Freedom	Sum of Squares	Mean Square	Percentage Variation	Percentage Variation (%)	*p*-Value
Among Provenances	18	686.448	38.136	0.715	3%	<0.01
Within Provenances	348	8565.051	24.612	24.612	97%	<0.01
Total	366	9251.499		25.327	100%	

**Table 5:** Geographic coordinates of the original US locations and numbers of *Robinia pseudoacacia* L. samples collected from each area.

	Provenance Name	Source Sites	Sample Number	Latitude (N)	Longitude (W)
	VA	Black Burg	12	37°26′	80°45′
		Independence	12	36°37′	81°07′
		Washington DC	14	38°27′	76°28′
	WV	New River Gorge	14	38°04′	81°03′
		Morgantown	11	39°36′	79°57′
		West Huntington	14	38°23′	82°29′
	NC	Huntersville	12	39°08′	90°45′
	GA	Blue Ridge Lake	13	34°51′	84°19′
	MD	Old National Pike	9	39°42′	78°18′
	PA	Big Beaver Blvd	13	40°32′	80°18′
		Bedford	15	40°08′	78°30′
	OH	Cadiz piedmont	12	40°07′	81°13′
		Cincinnati	10	39°03′	84°31′
	IN	Fisher	12	39°56′	85°53′
		Georgetwon	13	38°17′	85°55′
		Elberfeld	8	38°10′	87°26′
	IL	Bloomington	12	40°28′	89°01′
		Toe Exit	12	39°03′	88°40′
	KY	Mt Sterling	11	38°03′	84°02′
		Bowling Green	14	37°00′	86°17′
		Kentucky Lake	6	36°59′′	88°08′
		Wickliffe	10	37°01′	89°03′
	TN	Knoxville	13	35°52′	83°57′
		Waverly	14	35°53′	87°39′
	MS	Sardis	8	34°26′	89°53′
	AL	Upper Elkton RD	11	34°56′	86°53′
	MS/AL	Ms/AL Border	13	34°11′	88°06′
	IA	Brulington	10	40°50′	91°08′
	MO	Hannidal	8	39°43′	91°21′
		ST James	14	37°56′	91°08′
	KS	Riverton	4	37°06′	94°42′
	OK	Pryor	8	36°19′	95°18′
	AR	Colt	5	35°21′	80°51′
Total Number	19	33	367	-	-

“-” means no total number.

**Table 6:** Source of SSR primers in this study.

Type of primers	Number of Primers	Reference
EST-SSR	45	Guo et al. (2017) [48]
EST-SSR	25	Zhao K.Q. (2014) [47]
G-SSR	11	Mishima et al. (2008) [29]
G-SSR	3	Lian et al. (2004) [28]
G-SSR	7	Lian et al. (2002) [27]

## Supplementary Materials

Supplementary materials can be found at http://www.mdpi.com/1422-0067/19/9/2492/s1.
