# Supplementary material for: Evaluation of the Genetic Diversity and Differentiation of Black Locust (Robinia pseudoacacia L.) Based on Genomic and Expressed Sequence Tag-Simple Sequence Repeats
Source: Int J Mol Sci. 2018 Aug 23;19(9):2492. doi: 10.3390/ijms19092492 (PMC6164529; doi:10.3390/ijms19092492)
Supplement: Supplementary file 1 [file ijms-19-02492-s001.pdf]

Table S1 High- and stable- amplification SSR primers pairs used in this study

Primer Name		Sequencing information (5'-3')	Type of primers	Motif	Size	GenBank Accession No.
Rp-01	F	TGCAGAAAGAGAAAGCAGAGG	EST-SSR	$(TGTGAA)_4$	140	—
	R	CCGAACCCTTTCTGGTTAGTC				
Rp-02	F	GCTGCGTTTAATTTTGTCAGG	EST-SSR	$(GAAT)_4$	170	—
	R	TCAATCCATCAAAGAGGAAACA				
Rp-06	F	TGGACAAAACATCATCGTGTG	EST-SSR	$(TGAGTT)_4$	147	—
	R	CTCTCTTCTTTCTGCCCCTCA				
Rp-08	F	TCAGGTGCATAAGCTCATTACTTC	EST-SSR	$(AAAAT)_4$	152	—
	R	GGTTGTCAGATGAAATGCACA				
Rp-10	F	GGCATGTGGCTATGAAGATGT	EST-SSR	$(CCTTT)_4$	154	—
	R	TCAGTGGGACTTGGTTTCTTG				
Rp-11	F	GAAGCTATCACCGCAAATGAA	EST-SSR	$(AG)_{10}$	150	—
	R	GTCGAAGTGCGTCCTAGATCA				
Rp-12	F	AAGAGTCATCACGGAGACCAA	EST-SSR	$(AGCAGA)_4$	150	—
	R	GGAGTCCAATTAAGTGCGAGA				
Rp-13	F	CATTTCCGATTTCCAATTCCT	EST-SSR	$(CTCTTC)_4$	151	—
	R	GCCGAGGACTCGGTAGAAGT				
Rp-15	F	TTAACTAATGCGGCGAGAAGA	EST-SSR	$(TCAC)_5$	119	—
	R	GAGAGGAAGTGTGCGAAACAA				
Rp-19	F	CAGGAGTGGCAGCATTAGTGT	EST-SSR	$(AGGCTG)_4$	123	—
	R	CACAACAAGCACATTTTGCAC				
Rp-21	F	TATGATCACGTCCCCTAATGC	EST-SSR	$(CCA)_7$	146	—
	R	AAGTGGAAAGAAATGGGATGG				
Rp-22	F	GGTAAGGTGAAGGAGGTGGAG	EST-SSR	$(AGGGTT)_4$	150	—

	R	AGCTTGGTCTCCTAGGTCGTC				
Rp-23	F	GGAGGAGCAACCATCTGTGTA	EST-SSR	$(AGAAGT)_4$	146	—
	R	CTCCCTCTTCATCCTCACCTC				
Rp-28	F	CTTGGTCTAGAAAGTCCTGCT	EST-SSR	$(CAG)_7$	151	—
	R	GGTCATCAAGGTTAGTTGGAT				
Rp-30	F	TTGAACCAAAACTGGAAGAGC	EST-SSR	$(GCT)_8$	151	—
	R	GCACCGTACAGTTACCCTATCC				
Rp-31	F	GACCCCATTTTTCTCAAGGAC	EST-SSR	$(ATT)_7$	140	—
	R	TTGGATAAGTCGGTGAAGGTG				
Rp-33	F	CAAACAGTCTCATGGAAATGGA	EST-SSR	$(ATC)_7$	141	—
	R	GGGTTGGTATTGTTGGGAAAT				
Rp-35	F	TCAGACGTGGTAGAGCAGTGTT	EST-SSR	$(CACAC)_4$	152	—
	R	ATTTGTTTTTGGGGGAGATTG				
Rp-36	F	CGTTTCAGCCATTGATTTTGT	EST-SSR	$(GAATC)_5$	141	—
	R	GATCATCACCGTCCACCTTC				
Rp-37	F	TGTCGTCATTTTATTTTACCC	EST-SSR	$(GAACGA)_4$	152	—
	R	CTCACCCTTTTTATTTCCATT				
Rp-38	F	TCCATTCCCTGGTTTCTTCTT	EST-SSR	$(TC)_{10}$	150	—
	R	AGCACAATTTCCTCAGTGCAG				
Rp-40	F	TCATTGGACATCCCTCCATAA	EST-SSR	$(TAA)_8$	139	—
	R	GGCTCGACATGGTTGATTTT				
Rp-41	F	AACTCACCCAATTGCACACTC	EST-SSR	$(CCA)_7$	143	—
	R	GAGCAAGAGCTAAAGCAGCAA				
Rp-43	F	CAAAGCAGAGAGAATGTATGG	EST-SSR	$(CAAAT)_4$	155	—
	R	ATCCCTTGCTCCTTGTAATAG				
Rp-44	F	TATCTGGGAGAATCGAGAGCA	EST-SSR	$(ATCA)_5$	145	—

	R	CCACCATGGTTGTCCTTCTAA				
Rply1	F	AGTTCGCAAAGGAAGGAG	EST-SSR	$(AAG)_6$	243	—
	R	GGTAACAAGCACCAGCAA				
Rply2	F	TGTGAATGGTTGGTGGACAT	EST-SSR	$(CCA)_6$	161	—
	R	CGTTGCTTGGAGGAGAATAA				
Rply3	F	GCCTCATAAATAAAAGGAACG	EST-SSR	$(GTGGT)_4$	246	—
	R	CTGCCATTGGTAACTGGTAAA				
Rply5	F	GAGTCATGCCCTTTGTATGTT	EST-SSR	$(ATG)_8$	242	—
	R	TGTCACCTTCAAGTCCCTATT				
Rply15	F	GGCACAAGAACAGAACAAA	EST-SSR	$(CAT)_7$	239	—
	R	GTGGTGGATAAGGATAAGC				
Rply16	F	CGTCGGAGTGCTGTTATG	EST-SSR	$(GAA)_7$	242	—
	R	AGCTTATGGCAAAGAGGG				
Rply22	F	ATCACCATCTGTTCCTCCAC	EST-SSR	$(ACCTG)_4$	120	—
	R	TTCTCCTCAGCCACTTCTTT				
Rply28	F	TGGAGGGTTAGATTTGGATGT	EST-SSR	$(TGAGC)_4$	150	—
	R	AGGATGGAGAGTAAAGGGTTG				
Rply32	F	TGGGATCTTTGGAGGTAAT	EST-SSR	$(GTT)_5$	239	—
	R	TAGAGGCTCAATGGTTTCA				
Rply33	F	GCCCATTCTGTTGGTTCT	EST-SSR	$(ACT)_7$	266	—
	R	GGTTATGTGCCCTCGTGT				
Rply44	F	AAAGTGAGACATCCAAGTTC	EST-SSR	$(CT)_7$	153	—
	R	CAACCTTCCATTGTTTACAC				
Rply49	F	CCCCGTACAGTTCCATCT	EST-SSR	$(CT)_8$	120	—
	R	GACCTCGTAAAAGCCACC				
Rops02	F	CAGAACTGTGGAGAATAATTCTGAACCG	G-SSR	$(AC)_{13}(AT)_4$	107–138	AB075029

	R	CGCCATCTGTTAGTTTGTTGC				
Rops05	F	TGGTGATTAAGTCGCAAGGTG	G-SSR	$(AC)_2GC(AC)_7$	120–138	AB075031
	R	GTTGTGACTTGTACGTAAGTC				
Rops08	F	TTCTGAGGAAGGGTTCCGTGG	G-SSR	$(CA)_8TA(CA)_3$	192-212	AB075033
	R	GTTAAAGCAACAGGCACATGG				
Rops15	F	GCCCATTTTCAAGAATCCATATATTGG	G-SSR	$(CT)_{20}$	112–254	AB120731
	R	TCATCCTTGTTTTGGACAATC				
Rops16	F	AACCCTAAAAGCCTCGTTATC	G-SSR	$(CT)_{13}$	195–223	AB120732
	R	TGGCATTTTTTGGAAGACACC				
Rops18	F	AGATAAGATCAAGTGCAAGAGTGTAAG	G-SSR	$(AC)_8$	135–219	AB120733
	R	TAATCCTCGAGGGAACAATAC				
Rp01B	F	ACCAATTAGGTAACGTCAGC	G-SSR	$(CT)_{16}$	172–192	AB353937
	R	TGTTCACTGACAAAGCTG				
Rp032	F	GCATATTGCATATGCGCTTGTG	G-SSR	$(TG)_{13}$	109–135	AB353934
	R	TCCCTGAAGCTCATAACTGTCATGTG				
Rp106	F	AAACTGAATTATATCCCTTTACGGC	G-SSR	$(GT)_9$	143–154	AB353929
	R	GCATATATCCACCAGATACCCG				
Rp109	F	GAGGAATCACAAAACCGTTTGG	G-SSR	$(AG)_{17}$	119-151	AB353930
	R	TGGGATTTGAGAGAGTGGTGGTG				
Rp200	F	GGTTTCTTTGTTCACCTGCTCTGG	G-SSR	$(AG)_{23}$	160–198	AB353933
	R	ACCTACGTGTCCACGGCTCT				

Table S2 Analysis of genetic diversity for American black locus at 35 SSR loci

Locus	*N*	*Na*	*Ne*	*I*	*Ho*	*He*	F_{IS}	F_{IT}	F_{ST}	*Nm*	*PIC*	*H*	P_{HWE}
Rp-01	8	5.105	2.948	1.225	0.590	0.646	0.088	0.125	0.042	5.769	0.637	0.689	0.303
Rp-02	5	2.895	1.649	0.624	0.254	0.352	0.278	0.351	0.101	2.221	0.346	0.379	0.000
Rp-06	7	4.421	3.169	1.247	0.685	0.668	-0.027	0.049	0.074	3.126	0.689	0.732	0.364
Rp-10	10	4.053	1.926	0.814	0.408	0.438	0.067	0.160	0.100	2.248	0.447	0.495	0.493
Rp-12	12	5.211	3.290	1.308	0.722	0.681	-0.060	-0.017	0.040	5.934	0.664	0.712	0.205
Rp-13	4	2.947	1.378	0.490	0.232	0.259	0.102	0.157	0.061	3.821	0.270	0.286	0.594
Rp-15	8	3.789	1.503	0.589	0.309	0.294	-0.051	0.043	0.089	2.548	0.311	0.323	0.018
Rp-21	3	1.842	1.079	0.141	0.073	0.068	-0.078	-0.032	0.043	5.522	0.074	0.075	0.686
Rp-22	13	5.316	2.888	1.240	0.624	0.629	0.007	0.078	0.072	3.230	0.635	0.672	0.556
Rp-23	8	3.684	1.850	0.794	0.346	0.429	0.193	0.238	0.055	4.312	0.423	0.458	0.235
Rp-28	10	5.632	3.366	1.360	0.681	0.687	0.009	0.068	0.060	3.945	0.690	0.732	0.354
Rp-31	10	3.368	1.448	0.549	0.221	0.286	0.226	0.267	0.053	4.509	0.290	0.308	0.395
Rp-33	27	6.579	2.913	1.240	0.393	0.593	0.337	0.389	0.078	2.968	0.599	0.630	0.000
Rp-35	8	4.474	2.394	1.057	0.641	0.572	-0.121	-0.072	0.043	5.564	0.535	0.591	0.160
Rp-36	8	4.368	2.897	1.179	0.712	0.648	-0.098	-0.042	0.051	4.653	0.617	0.677	0.371
Rp-37	13	5.263	2.998	1.261	0.470	0.649	0.276	0.329	0.073	3.166	0.636	0.679	0.269
Rp-38	19	8.421	5.081	1.794	0.835	0.792	-0.053	0.004	0.054	4.371	0.820	0.838	0.411
Rp-41	8	3.947	1.552	0.636	0.297	0.315	0.057	0.129	0.077	2.992	0.319	0.329	0.611
Rp-43	4	3.105	1.991	0.819	0.552	0.479	-0.151	-0.095	0.049	4.883	0.450	0.505	0.041
Rp-44	5	1.842	1.065	0.119	0.044	0.054	0.190	0.231	0.050	4.766	0.061	0.061	0.029
Rply2	11	5.632	3.840	1.454	0.663	0.732	0.094	0.144	0.055	4.296	0.745	0.779	0.329
Rply5	8	4.737	2.743	1.181	0.571	0.623	0.084	0.124	0.044	5.463	0.613	0.656	0.191
Rply15	12	5.105	2.454	1.090	0.419	0.548	0.236	0.316	0.105	2.133	0.584	0.614	0.147
Rply16	20	6.579	1.922	0.989	0.417	0.442	0.056	0.105	0.051	4.620	0.487	0.497	0.680

Rply22	8	5.105	2.949	1.226	0.593	0.646	0.082	0.121	0.043	5.620	0.638	0.690	0.313
Rply28	8	3.211	1.917	0.798	0.373	0.451	0.173	0.214	0.050	4.751	0.431	0.475	0.329
Rply33	10	3.474	1.390	0.509	0.164	0.253	0.353	0.393	0.063	3.711	0.280	0.292	0.273
Rply49	26	10.158	4.573	1.789	0.556	0.752	0.261	0.318	0.077	3.010	0.798	0.812	0.128
Rops05	25	10.211	6.551	2.036	0.602	0.837	0.281	0.329	0.068	3.448	0.886	0.895	0.000
Rops08	14	6.368	3.258	1.360	0.596	0.652	0.086	0.186	0.110	2.027	0.721	0.746	0.405
Rops18	34	10.368	6.552	1.999	0.551	0.830	0.336	0.391	0.082	2.795	0.898	0.906	0.068
Rp01B	17	8.368	4.735	1.740	0.699	0.772	0.094	0.156	0.069	3.351	0.816	0.832	0.003
Rp032	18	7.684	5.303	1.758	0.492	0.787	0.375	0.430	0.089	2.549	0.852	0.866	0.000
Rp106	12	4.947	3.372	1.326	0.719	0.695	-0.035	0.021	0.054	4.392	0.685	0.732	0.268
Rp109	26	10.105	5.631	1.910	0.810	0.813	0.004	0.075	0.071	3.270	0.858	0.871	0.346
Mean	12.543	5.380	2.988	1.133	0.495	0.553	0.105	0.162	0.066	3.885	0.566	0.595	0.274
Total	439												

N: Number of alleles per locus; N_a: Number of different alleles; *Ne*: Effective number of alleles; *I*: Shannon's Information index; *Ho*: Observed heterozygosity; *He*: Expected heterozygosity; F_{IS}: Inbreeding coefficient; F_{IT}: Inbreeding within entire provenance; F_{ST}: Fixation index; *Nm*: Gene flow; *PIC*: Polymorphism information content; *H*: Gene Diversity.

Table S3 Average gene diversity index values for 19 provenances of *Robinia pseudoacacia* L. (Standard Error (SE) values in brackets)

Provenance	*Na*	*Na* Freq. ≤ 5%	*Ne*	*I*	No. Private Alleles	No. Lcomm Alleles (≤25%)	No. Lcomm Alleles (≤50%)	*He*
VA	7.229	3.571	3.170	1.231	0.171	1.543	2.686	0.572
(Washington)	(0.731)	(0.273)	(0.342)	(0.100)	(0.077)	(0.302)	(0.449)	(0.038)
WV	7.629	3.829	3.382	1.304	0.343	1.457	2.686	0.594
(West Virginia)	(0.656)	(0.283)	(0.349)	(0.100)	(0.136)	(0.257)	(0.362)	(0.038)
NC	4.629	3.314	2.795	1.055	0.029	0.686	1.400	0.525
(North Carolina)	(0.402)	(0.289)	(0.270)	(0.097)	(0.029)	(0.178)	(0.225)	(0.041)
GA	4.857	3.343	2.888	1.093	0.057	0.486	1.229	0.543
(Georgia)	(0.367)	(0.301)	(0.276)	(0.092)	(0.040)	(0.138)	(0.225)	(0.040)
MD	4.200	4.200	2.781	1.045	0.086	0.543	1.000	0.536
(Maryland)	(0.338)	(0.338)	(0.240)	(0.090)	(0.063)	(0.138)	(0.183)	(0.040)
PA	6.143	3.571	2.992	1.173	0.229	1.057	2.200	0.559
(Pennsylvania)	(0.586)	(0.294)	(0.266)	(0.099)	(0.072)	(0.224)	(0.359)	(0.040)
OH	6.029	3.743	3.337	1.223	0.229	1.086	2.200	0.573
(Ohio)	(0.551)	(0.308)	(0.343)	(0.106)	(0.083)	(0.198)	(0.322)	(0.042)
IN	6.857	3.857	3.527	1.306	0.114	1.514	2.771	0.604
(Indiana)	(0.634)	(0.307)	(0.361)	(0.102)	(0.068)	(0.247)	(0.406)	(0.039)
IL	5.943	3.371	3.037	1.162	0.057	1.200	2.200	0.552
(Illinois)	(0.505)	(0.256)	(0.334)	(0.096)	(0.040)	(0.196)	(0.327)	(0.039)
KY	7.400	3.714	3.308	1.302	0.143	1.629	2.886	0.599
(Kentucky)	(0.635)	(0.248)	(0.313)	(0.095)	(0.073)	(0.275)	(0.441)	(0.037)
TN	6.229	4.029	3.208	1.236	0.143	1.000	2.029	0.588
(Tennessee)	(0.511)	(0.305)	(0.296)	(0.092)	(0.060)	(0.225)	(0.327)	(0.037)
MS	3.743	3.743	2.588	0.953	0.086	0.543	0.943	0.506
(Mississippi)	(0.318)	(0.318)	(0.217)	(0.090)	(0.048)	(0.125)	(0.183)	(0.042)
AL	4.714	3.457	2.846	1.074	0.086	0.886	1.486	0.528
(Alabama)	(0.397)	(0.308)	(0.289)	(0.096)	(0.048)	(0.196)	(0.285)	(0.041)
MS/AL	4.743	3.514	2.801	1.104	0.086	0.686	1.314	0.552
(Mississippi/Alabama)	(0.305)	(0.240)	(0.223)	(0.082)	(0.048)	(0.152)	(0.182)	(0.037)
IA	4.314	4.314	2.764	1.052	0.086	0.714	1.314	0.533
(Iowa)	(0.325)	(0.325)	(0.263)	(0.087)	(0.048)	(0.151)	(0.245)	(0.039)
MO	6.314	3.314	3.031	1.190	0.114	1.229	2.429	0.558
(Missouri)	(0.518)	(0.286)	(0.286)	(0.099)	(0.055)	(0.205)	(0.353)	(0.040)
KS	3.086	3.086	2.457	0.875	0.086	0.200	0.543	0.487
(Kansas)	(0.244)	(0.244)	(0.209)	(0.085)	(0.063)	(0.090)	(0.150)	(0.043)
OK	4.543	4.543	3.148	1.149	0.143	0.771	1.371	0.577
(Oklahoma)	(0.349)	(0.349)	(0.290)	(0.093)	(0.073)	(0.174)	(0.225)	(0.042)
AR	3.629	3.629	2.708	0.999	0.029	0.429	1.000	0.530
(Arkansas)	(0.269)	(0.269)	(0.209)	(0.089)	(0.029)	(0.125)	(0.213)	(0.043)
Mean	5.380	3.692	2.988	1.133	0.122	0.929	1.773	0.553
	(0.455)	(0.292)	(0.283)	(0.094)	(0.061)	(0.189)	(0.288)	(0.042)

Na: No. of Different Alleles; *Na* Freq. ≥ 5%: No. of different alleles with a frequency ≥ 5%; *Ne*: No. of effective alleles = 1/(Sum pi^2); *I*: Shannon's Information Index, *I* = −1·Sum (pi·Ln (pi)); No. Private Alleles: No. of alleles unique to a single provenance; No. LComm Alleles (≤25%): No. of locally common alleles (Freq. ≥ 5%) found in 25% or fewer provenances; No. Lcomm Alleles (≤50%): No. of locally common alleles (Freq. ≥ 5%) Found in 50% or fewer provenances; *He*: Expected heterozygosity, *He* = 1 – Sum pi^2

Table S4 Summary of private alleles by provenance

Provenance	Locus	Allele	Frequence
VA	Rp-31	132	0.014
VA	Rply2	144	0.014
VA	Rply16	248	0.013
VA	Rops08	202	0.015
VA	Rops18	143	0.014
VA	Rops18	240	0.027
WV	Rp-15	108	0.013
WV	Rp-33	139	0.029
WV	Rp-33	187	0.014
WV	Rp-37	139	0.013
WV	Rply15	103	0.013
WV	Rops08	210	0.013
WV	Rops18	148	0.015
WV	Rops18	180	0.015
WV	Rops18	182	0.030
WV	Rops18	184	0.030
WV	Rp01B	164	0.014
WV	Rp109	107	0.013
NC	Rp-33	153	0.111
GA	Rp-10	154	0.038
GA	Rp-12	178	0.038
MD	Rply49	120	0.056
MD	Rply49	122	0.056
MD	Rp01B	168	0.111
PA	Rp-15	120	0.018
PA	Rp-28	156	0.036
PA	Rply33	259	0.018
PA	Rply49	166	0.018
PA	Rops08	229	0.019
PA	Rops18	155	0.024
PA	Rp032	91	0.056
PA	Rp109	135	0.018
OH	Rp-12	136	0.023
OH	Rp-41	163	0.136
OH	Rply49	108	0.023
OH	Rops05	127	0.024
OH	Rops05	129	0.024
OH	Rops08	232	0.029
OH	Rp01B	200	0.024
OH	Rp032	129	0.025
IN	Rp-22	204	0.016
IN	Rp-33	145	0.015

IN	Rp-33	175	0.015
IN	Rops08	227	0.016
IL	Rp-38	140	0.023
IL	Rp109	111	0.042
KY	Rp-23	174	0.012
KY	Rp-44	145	0.012
KY	Rply28	137	0.012
KY	Rply28	191	0.024
KY	Rops18	244	0.024
TN	Rp-06	110	0.037
TN	Rp-37	133	0.038
TN	Rply16	287	0.019
TN	Rops18	152	0.019
TN	Rp106	147	0.019
MS	Rp-37	199	0.063
MS	Rply33	274	0.063
MS	Rops18	224	0.063
AL	Rp-36	139	0.045
AL	Rply49	116	0.045
AL	Rp106	159	0.045
MS/AL	Rply28	131	0.077
MS/AL	Rp106	137	0.077
MS/AL	Rp109	105	0.038
IA	Rp-10	189	0.100
IA	Rp-36	129	0.050
IA	Rply16	290	0.050
MO	Rp-28	149	0.023
MO	Rply33	238	0.023
MO	Rops18	147	0.029
MO	Rp032	95	0.059
KS	Rp-22	154	0.125
KS	Rp-22	160	0.125
KS	Rp-33	148	0.333
OK	Rp-31	129	0.063
OK	Rp-31	135	0.063
OK	Rp-35	132	0.125
OK	Rp-37	205	0.063
OK	Rp109	115	0.188
AR	Rp109	117	0.100

Table S5. Pairwise F_{ST}（lower diagonal）and significance test (upper diagonal) of all black locust samples.

	**VA (Washington)**	**WV (West Virginia)**	**NC (North Carolina)**	**GA (Georgia)**	**MD (Maryland)**	**PA (Pennsylvania)**	**OH (Ohio)**	**IN (Indiana)**	**IL (Illinois)**	**KY (Kentucky)**	**TN (Tennessee)**	**MS (Mississippi)**	**AL (Alabama)**	**MS/AL (Mississippi/Alabama)**	**IA (Iowa)**	**MO (Missouri)**	**KS (Kansas)**	**OK (Oklahoma)**	**AR (Arkansas)**
VA (Washington)		*	*	*	*	*	*	*	*	*	*	*	*	*	NS	*	NS	*	*
WV (West Virginia)	0.011		NS	*	NS	NS	*	*	*	NS	*	*	*	*	NS	*	NS	*	NS
NC (North Carolina)	0.021	0.022		*	*	NS	*	*	*	NS	*	*	*	NS	NS	NS	NS	*	NS
GA (Georgia)	0.027	0.029	0.045		*	*	*	*	*	*	*	*	*	*	*	*	NS	*	NS
MD (Maryland)	0.031	0.024	0.047	0.060		*	*	*	NS	NS	*	*	*	*	NS	*	NS	*	*
PA (Pennsylvania)	0.013	0.011	0.022	0.033	0.033		*	*	*	*	*	*	*	*	*	*	NS	*	NS
OH (Ohio)	0.018	0.015	0.030	0.035	0.038	0.020		*	*	*	*	*	*	*	NS	*	NS	*	NS
IN (Indiana)	0.016	0.011	0.025	0.036	0.034	0.017	0.017		*	NS	*	*	*	*	*	*	NS	*	NS
IL (Illinois)	0.015	0.012	0.023	0.036	0.033	0.016	0.020	0.018		*	*	*	*	*	*	NS	NS	*	*
KY (Kentucky)	0.013	0.007	0.021	0.033	0.027	0.013	0.018	0.009	0.014		*	*	*	*	*	*	NS	*	NS
TN (Tennessee)	0.015	0.015	0.030	0.032	0.037	0.020	0.019	0.016	0.019	0.012		*	*	*	*	*	NS	*	NS
MS (Mississippi)	0.043	0.037	0.051	0.071	0.058	0.043	0.041	0.041	0.042	0.038	0.044		*	*	*	*	*	*	*
AL (Alabama)	0.032	0.025	0.042	0.046	0.055	0.033	0.029	0.031	0.031	0.027	0.029	0.052		*	*	*	NS	*	NS

MS/AL (Mississippi/Alabama)	0.031	0.026	0.036	0.051	0.054	0.034	0.032	0.029	0.035	0.025	0.027	0.063	0.039		*	*	NS	*	NS
IA (Iowa)	0.023	0.022	0.030	0.049	0.041	0.026	0.026	0.027	0.025	0.023	0.030	0.054	0.042	0.038		NS	NS	*	NS
MO (Missouri)	0.020	0.016	0.022	0.044	0.034	0.020	0.022	0.017	0.015	0.014	0.021	0.038	0.029	0.032	0.023		NS	*	NS
KS (Kansas)	0.044	0.043	0.049	0.073	0.067	0.042	0.054	0.053	0.041	0.045	0.055	0.081	0.069	0.062	0.055	0.045		NS	NS
OK (Oklahoma)	0.036	0.033	0.049	0.053	0.065	0.038	0.040	0.034	0.038	0.032	0.037	0.069	0.050	0.044	0.056	0.043	0.073		NS
AR (Arkansas)	0.040	0.038	0.047	0.056	0.066	0.046	0.038	0.039	0.045	0.039	0.040	0.069	0.044	0.044	0.050	0.041	0.089	0.055	

* $p < 0.05$; NS: Not significant.

Table S6. Nei's unbiased genetic distance (lower diagonal) and genetic identity (upper diagonal) among black locust provenances.

	VA	WV	NC	GA	MD	PA	OH	IN	IL	KY	TN	MS	AL	MS/AL	IA	MO	KS	OK	AR
VA		0.988	0.979	0.958	0.967	0.986	0.973	0.974	0.979	0.980	0.983	0.937	0.946	0.947	0.981	0.969	0.976	0.946	0.970
WV	0.012		0.978	0.953	0.987	0.991	0.983	0.990	0.988	0.998	0.983	0.953	0.971	0.961	0.984	0.981	0.987	0.954	0.975
NC	0.021	0.022		0.927	0.954	0.981	0.963	0.973	0.976	0.982	0.965	0.935	0.942	0.961	0.980	0.981	0.986	0.947	0.985
GA	0.043	0.048	0.076		0.905	0.942	0.933	0.938	0.935	0.939	0.950	0.868	0.920	0.901	0.923	0.918	0.922	0.913	0.937
MD	0.033	0.014	0.047	0.100		0.966	0.959	0.962	0.963	0.977	0.955	0.935	0.926	0.920	0.966	0.970	0.957	0.904	0.943
PA	0.015	0.009	0.019	0.060	0.035		0.973	0.981	0.980	0.988	0.973	0.936	0.950	0.943	0.977	0.975	0.993	0.945	0.963
OH	0.027	0.017	0.038	0.069	0.042	0.027		0.982	0.973	0.975	0.971	0.946	0.957	0.944	0.980	0.969	0.965	0.939	0.976
IN	0.027	0.010	0.027	0.065	0.038	0.019	0.018		0.975	0.996	0.980	0.944	0.955	0.952	0.970	0.978	0.955	0.955	0.972
IL	0.021	0.012	0.024	0.067	0.038	0.020	0.028	0.026		0.985	0.976	0.941	0.954	0.942	0.976	0.987	0.991	0.949	0.960
KY	0.021	0.002	0.018	0.063	0.023	0.012	0.026	0.004	0.016		0.987	0.947	0.963	0.961	0.979	0.985	0.972	0.960	0.973
TN	0.017	0.017	0.036	0.052	0.046	0.027	0.029	0.020	0.025	0.013		0.934	0.960	0.958	0.966	0.969	0.957	0.949	0.971
MS	0.065	0.048	0.067	0.141	0.068	0.066	0.055	0.058	0.061	0.054	0.068		0.926	0.897	0.927	0.948	0.926	0.901	0.932
AL	0.055	0.029	0.060	0.083	0.077	0.051	0.044	0.046	0.047	0.038	0.041	0.077		0.943	0.946	0.963	0.935	0.936	0.975
MS/AL	0.055	0.040	0.039	0.104	0.083	0.059	0.058	0.049	0.060	0.040	0.043	0.108	0.058		0.955	0.949	0.954	0.945	0.963
IA	0.019	0.016	0.020	0.081	0.035	0.023	0.020	0.030	0.024	0.021	0.035	0.076	0.055	0.046		0.987	0.983	0.927	0.973
MO	0.031	0.019	0.019	0.085	0.030	0.025	0.031	0.023	0.013	0.016	0.032	0.053	0.037	0.052	0.013		0.980	0.941	0.972
KS	0.024	0.013	0.015	0.081	0.044	0.007	0.036	0.046	0.009	0.029	0.044	0.076	0.067	0.047	0.017	0.020		0.950	0.930
OK	0.056	0.048	0.054	0.091	0.101	0.056	0.062	0.046	0.052	0.041	0.053	0.104	0.066	0.057	0.076	0.061	0.051		0.966
AR	0.030	0.025	0.015	0.065	0.058	0.038	0.025	0.029	0.041	0.028	0.029	0.070	0.025	0.037	0.027	0.028	0.072	0.035	

Table S7 Overview of 367 *Robinia pseudoacacia* L. samples and their sources

**Sample No.**	**Source Site**	**Latitude (E)**	**Longitudes (N)**	**Sample No.**	**Source Site**	**Latitude (E)**	**Longitudes (N)**
MJ-1	Black Burg VA	34°49.302′	112°28,103′	MJ-9	New River Gorge WV	34°49.295′	112°28,141′
MJ-2	Black Burg VA	34°49.304′	112°28,105′	MJ-10	New River Gorge WV	34°49.294′	112°28,148′
MJ-3	Black Burg VA	34°49.298′	112°28,110′	MJ-11	New River Gorge WV	34°49.294′	112°28,155′
MJ-4	Black Burg VA	34°49.297′	112°28,116′	MJ-12	New River Gorge WV	34°49.291′	112°28,158′
MJ-5	Black Burg VA	34°49.297′	112°28,123′	MJ-13	New River Gorge WV	34°49.292′	112°28,164′
MJ-6	Black Burg VA	34°49.297′	112°28,127′	MQ-7	New River Gorge WV	34°43.984′	115°05,138′
MQ-1	Black Burg VA	34°43.958′	115°05,134′	MQ-8	New River Gorge WV	34°43.994′	115°05,138′
MQ-2	Black Burg VA	34°43.960′	115°05,138′	MQ-9	New River Gorge WV	34°43.990′	115°05,142′
MQ-3	Black Burg VA	34°43.970′	115°05,136′	MQ-10	New River Gorge WV	34°43.998′	115°05,141′
MQ-4	Black Burg VA	34°43.978′	115°05,135′	MQ-11	New River Gorge WV	34°44.001′	115°05,143′
MQ-5	Black Burg VA	34°43.980′	115°05,132′	MQ-12	New River Gorge WV	34°44.010′	115°05,147′
MQ-6	BlackBurg VA	34°43.980′	115°05,136′	MQ-13	New River Gorge WV	34°44.014′	115°05,148′
MJ-135	Independence VA	34°49.322′	115°28,116′	MJ-61	Morgantown WV	34°49.311′	115°28,137′
MJ-136	Independence VA	34°49.318′	115°28,111′	MJ-62	Morgantown WV	34°49.308′	115°28,141′
MJ-137	Independence VA	34°49.320′	115°28,109′	MJ-63	Morgantown WV	34°49.308′	115°28,143′
MJ-138	Independence VA	34°49.320′	115°28,101′	MJ-64	Morgantown WV	34°49.308′	115°28,153′
MJ-139	Independence VA	34°49.322′	115°28,108′	MJ-65	Morgantown WV	34°49.306′	115°28,158′
MJ-140	Independence VA	34°49.323′	115°28,111′	MJ-66	Morgantown WV	34°49.307′	115°28,162′
MQ-121	Independence VA	34°44.004′	115°05,165′	MQ-72	Morgantown WV	34°44.041′	115°05,157′
MQ-120	Independence VA	34°44.009′	115°05,166′	MQ-71	Morgantown WV	34°44.035′	115°05,158′
MQ-119	Independence VA	34°44.009′	115°05,169′	MQ-70	Morgantown WV	34°44.030′	115°05,160′
MQ-118	Independence VA	34°44.046′	115°05,178′	MQ-69	Morgantown WV	34°44.026′	115°05,159′
MQ-117	Independence VA	34°44.017′	115°05,165′	MQ-68	Morgantown WV	34°44.022′	115°05,155′
MQ-116	Independence VA	34°44.024′	115°05,173′	MJ-67	West Huntington WV	34°49.305′	115°28,168′
MJ-186	Washington DC	34°49.290′	115°28,306′	MJ-68	West Huntington WV	34°49.302′	115°28,173′
MJ-187	Washington DC	34°49.291′	115°28,298′	MJ-69	West Huntington WV	34°49.303′	115°28,179′
MJ-188	Washington DC	34°49.292′	115°28,289′	MJ-70	West Huntington WV	34°49.303′	115°28,181′
MJ-189	Washington DC	34°49.292′	115°28,289′	MJ-71	West Huntington WV	34°49.301′	115°28,191′
MJ-190	Washington DC	34°49.292′	115°28,278′	MJ-72	West Huntington WV	34°49.295′	115°28,209′
MJ-191	Washington DC	34°49.293′	115°28,272′	MJ-73	West Huntington WV	34°49.293′	115°28,214′
MJ-192	Washington DC	34°49.292′	115°28,269′	MQ-66	West Huntington WV	34°44.009‘	115°05,160′
MQ-183	Washington DC	34°44.041′	115°05,180′	MQ-65	West Huntington WV	34°44.007′	115°05,156′
MQ-182	Washington DC	34°44.032′	115°05,181′	MQ-64	West Huntington WV	34°44.005′	115°05,154′
MQ-181	Washington DC	34°44.028′	115°05,178′	MQ-63	West Huntington WV	34°44.000′	115°05,152′
MQ-180	Washington DC	34°44.026′	115°05,174′	MQ-62	West Huntington WV	34°43.998′	115°05,151′
MQ-179	Washington DC	34°44.016′	115°05,176′	MQ-61	West Huntington WV	34°43.987′	115°05,149′
MQ-178	Washington DC	34°44.013′	115°05,177′	MQ-60	West Huntington WV	34°43.985′	115°05,152′
MQ-177	Washington DC	34°44.007′	115°05,175′	MJ-141	Huntersille NC	34°49.323′	115°28,117′
MJ-7	New River Gorge WV	34°49.296′	112°28,134′	MJ-142	Huntersille NC	34°49.320′	115°28,124′
MJ-8	New River Gorge WV	34°49.293′	112°28,142′	MJ-143	Huntersille NC	34°49.323′	115°28,125′
MJ-144	Huntersille NC	34°49.320′	115°28,130′	MJ-54	Bedford PA	34°49.311′	115°28,100′

MJ-145	Huntersille NC	34°49.317′	115°28,139′	MJ-55	Bedford PA	34°49.316′	115°28,107′
MJ-146	Huntersille NC	34°49.320′	115°28,143′	MJ-56	Bedford PA	34°49.314′	115°28,109′
MQ-115	Huntersille NC	34°44.027′	115°05,170′	MJ-57	Bedford PA	34°49.313′	115°28,117′
MQ-114	Huntersille NC	34°44.034′	115°05,172′	MJ-58	Bedford PA	34°49.314′	115°28,123′
MQ-113	Huntersille NC	34°44.036′	115°05,169′	MJ-59	Bedford PA	34°49.311′	115°28,126′
MQ-112	Huntersille NC	34°44.040′	115°05,169′	MJ-60	Bedford PA	34°49.310′	115°28,127′
MQ-111	Huntersille NC	34°44.043′	115°05,170′	MQ-48	Bedford PA	34°44.008′	115°05,150′
MQ-147	Huntersille NC	34°44.033′	115°05,174′	MQ-49	Bedford PA	34°44.011′	115°05,149′
MJ-147	Blue Ridge Lake GA	34°49.315′	115°28,147′	MQ-50	Bedford PA	34°44.014′	115°05,150′
MJ-148	Blue Ridge Lake GA	34°49.316′	115°28,156′	MQ-51	Bedford PA	34°44.022′	115°05,150′
MJ-149	Blue Ridge Lake GA	34°49.312′	115°28,165′	MQ-198	Bedford PA	34°44.010′	115°05,200′
MJ-150	Blue Ridge Lake GA	34°49.311′	115°28,168′	MQ-52	Bedford PA	34°44.031′	115°05,153′
MJ-151	Blue Ridge Lake GA	34°49.312′	115°28,172′	MQ-53	Bedford PA	34°44.036′	115°05,154′
MJ-152	Blue Ridge Lake GA	34°49.311′	115°28,180′	MQ-54	Bedford PA	34°44.037′	115°05,156′
MJ-153	Blue Ridge Lake GA	34°49.311′	115°28,186′	MJ-21	Cadiz piedmont OH	34°49.303′	115°28,135′
MQ-146	Blue Ridge Lake GA	34°44.018′	115°05,172′	MJ-22	Cadiz piedmont OH	34°49.304′	115°28,127′
MQ-145	Blue Ridge Lake GA	34°44.022′	115°05,171′	MJ-23	Cadiz piedmont OH	34°49.305′	115°28,126′
MQ-144	Blue Ridge Lake GA	34°44.022′	115°05,175′	MJ-24	Cadiz piedmont OH	34°49.304′	115°28,124′
MQ-143	Blue Ridge Lake GA	34°44.013′	115°05,171′	MJ-25	Cadiz piedmont OH	34°49.306′	115°28,119′
MQ-142	Blue Ridge Lake GA	34°44.012′	115°05,168′	MJ-26	Cadiz piedmont OH	34°49.306′	115°28,114′
MQ-141	Blue Ridge Lake GA	34°44.006′	115°05,172′	MQ-19	Cadiz piedmont OH	34°44.038′	115°05,156′
MJ-49	Old National Pike MD	34°49.311′	115°28,131′	MQ-20	Cadiz piedmont OH	34°44.033′	115°05,153′
MJ-50	Old National Pike MD	34°49.309′	115°28,119′	MQ-21	Cadiz piedmont OH	34°44.031′	115°05,150′
MJ-51	Old National Pike MD	34°49.310′	115°28,120′	MQ-22	Cadiz piedmont OH	34°44.029′	115°05,156′
MJ-52	Old National Pike MD	34°49.312′	115°28,116′	MQ-23	Cadiz piedmont OH	34°44.023′	115°05,157′
MJ-53	Old National Pike MD	34°49.312′	115°28,110′	MQ-24	Cadiz piedmont OH	34°44.015′	115°05,153′
MQ-44	Old National Pike MD	34°43.992′	115°05,150′	MJ-161	Cincinati OH	34°49.302′	115°28,231′
MQ-45	Old National Pike MD	34°43.996′	115°05,150′	MJ-162	Cincinati OH	34°49.303′	115°28,231′
MQ-46	Old National Pike MD	34°44.001′	115°05,150′	MJ-163	Cincinati OH	34°49.301′	115°28,234′
MQ-47	Old National Pike MD	34°44.005′	115°05,150′	MJ-164	Cincinati OH	34°49.303′	115°28,237′
MJ-14	Big Beaver Blvd PA	34°49.296′	112°28,167′	MJ-165	Cincinati OH	34°49.302′	115°28,239′
MJ-15	Big Beaver Blvd PA	34°49.296′	112°28,162′	MQ-134	Cincinati OH	34°43.971′	115°05,161′
MJ-16	Big Beaver Blvd PA	34°49.299′	112°28,159′	MQ-133	Cincinati OH	34°43.968′	115°05,164′
MJ-17	Big Beaver Blvd PA	34°49.298′	112°28,156′	MQ-132	Cincinati OH	34°43.960′	115°05,159′
MJ-18	Big Beaver Blvd PA	34°49.300′	115°28,148′	MQ-131	Cincinati OH	34°43.963′	115°05,161′
MJ-19	Big Beaver Blvd PA	34°49.300′	115°28,147′	MQ-130	Cincinati OH	34°43.959′	115°05,164′
MJ-20	Big Beaver Blvd PA	34°49.301′	115°28,140′	MJ-27	Fisher IN	34°49.308′	115°28,109′
MQ-14	Big Beaver Blvd PA	34°44.012′	115°05,144′	MJ-28	Fisher IN	34°49.307′	115°28,102′
MQ-15	Big Beaver Blvd PA	34°44.022′	115°05,145′	MJ-29	Fisher IN	34°49.311′	115°28,103′
MQ-16	Big Beaver Blvd PA	34°44.026′	115°05,145′	MJ-30	Fisher IN	34°49.311′	115°28,108′
MQ-17	Big Beaver Blvd PA	34°44.029′	115°05,154′	MJ-31	Fisher IN	34°49.308′	115°28,114′
MQ-18	Big Beaver Blvd PA	34°44.031′	115°05,146′	MJ-32	Fisher IN	34°49.306′	115°28,119′
MQ-184	Big Beaver Blvd PA	34°44.029′	115°05,184′	MQ-25	Fisher IN	34°44.010′	115°05,152′
MQ-26	Fisher IN	34°44.0122′	115°05,146′	MQ-75	Toe Exit IL	34°44.031′	115°05,163′

MQ-27	Fisher IN	34°43.009′	115°05,146′	MQ-74	Toe Exit IL	34°44.037′	115°05,162′
MQ-28	Fisher IN	34°43.999′	115°05,148′	MQ-73	Toe Exit IL	34°44.039′	115°05,160′
MQ-29	Fisher IN	34°43.993′	115°05,151′	MQ-110	Toe Exit IL	34°44.038′	115°05,170′
MQ-30	Fisher IN	34°43.987′	115°05,151′	MQ-109	Toe Exit IL	34°44.034′	115°05,169′
MJ-80	Georegetwon IN	34°49.295′	115°28,248′	MQ-108	Toe Exit IL	34°44.028′	115°05,167′
MJ-81	Georegetwon IN	34°49.288′	115°28,254′	MJ-74	Mt sterling KY	34°49.293′	115°28,216′
MJ-82	Georegetwon IN	34°49.288′	115°28,256′	MJ-75	Mt sterling KY	34°49.293′	115°28,223′
MJ-83	Georegetwon IN	34°49.286′	115°28,264′	MJ-76	Mt sterling KY	34°49.295′	115°28,231′
MJ-84	Georegetwon IN	34°49.287′	115°28,268′	MJ-77	Mt sterling KY	34°49.295′	115°28,234′
MJ-85	Georegetwon IN	34°49.283′	115°28,276′	MJ-78	Mt sterling KY	34°49.296′	115°28,238′
MJ-86	Georegetwon IN	34°49.280′	115°28,282′	MJ-79	Mt sterling KY	34°49.295′	115°28,241′
MQ-55	Georegetwon IN	34°43.957′	115°05,143′	MQ-59	Mt sterling KY	34°43.979′	115°05,144′
MQ-91	Georegetwon IN	34°43.964′	115°05,151′	MQ-58	Mt sterling KY	34°43.977′	115°05,152′
MQ-90	Georegetwon IN	34°43.967′	115°05,149′	MQ-57	Mt sterling KY	34°43.972′	115°05,148′
MQ-89	Georegetwon IN	34°43.972′	115°05,149′	MQ-197	Mt sterling KY	34°43.996′	115°05,180′
MQ-88	Georegetwon IN	34°43.976′	115°05,149′	MQ-56	Mt sterling KY	34°43.960′	115°05,147′
MQ-87	Georegetwon IN	34°43.980′	115°05,150′	MJ-113	Bowling Green KY	34°49.298′	115°28,227′
MJ-88	Elberfeld IN	34°49.280′	115°28,294′	MJ-114	Bowling Green KY	34°49.300′	115°28,226′
MJ-89	Elberfeld IN	34°49.279′	115°28,293′	MJ-115	Bowling Green KY	34°49.297′	115°28,224′
MJ-90	Elberfeld IN	34°49.278′	115°28,303′	MJ-116	Bowling Green KY	34°49.303′	115°28,221′
MJ-91	Elberfeld IN	34°49.277′	115°28,300′	MJ-117	Bowling Green KY	34°49.298′	115°28,213′
MQ-85	Elberfeld IN	34°43.990′	115°05,150′	MJ-118	Bowling Green KY	34°49.300′	115°28,210′
MQ-84	Elberfeld IN	34°43.992′	115°05,152′	MJ-119	Bowling Green KY	34°49.302′	115°28,208′
MQ-86	Elberfeld IN	34°43.984′	115°05,149′	MQ-103	Bowling Green KY	34°44.009′	115°05,165′
MQ-83	Elberfeld IN	34°43.995′	115°05,153′	MQ-102	Bowling Green KY	34°44.002′	115°05,166′
MJ-33	Bloomington IL	34°49.305′	115°28,127′	MQ-101	Bowling Green KY	34°43.999′	115°05,161′
MJ-34	Bloomington IL	34°49.304′	115°28,130′	MQ-100	Bowling Green KY	34°43.998′	115°05,161′
MJ-35	Bloomington IL	34°49.304′	115°28,136′	MQ-99	Bowling Green KY	34°43.991′	115°05,162′
MJ-36	Bloomington IL	34°49.301′	115°28,141′	MQ-98	Bowling Green KY	34°43.984′	115°05,154′
MJ-37	Bloomington IL	34°49.302′	115°28,149′	MQ-97	Bowling Green KY	34°43.979′	115°05,159′
MJ-38	Bloomington IL	34°49.300′	115°28,154′	MQ-165	Kenturky Lake KY	34°43.960′	115°05,164′
MQ-31	Bloomington IL	34°43.984′	115°05,149′	MQ-164	Kenturky Lake KY	34°43.965′	115°05,165′
MQ-32	Bloomington IL	34°43.980′	115°05,140′	MJ-166	Kenturky Lake KY	34°49.300′	115°28,243′
MQ-33	Bloomington IL	34°43.974′	115°05,137′	MJ-167	Kenturky Lake KY	34°49.303′	115°28,248′
MQ-34	Bloomington IL	34°43.968′	115°05,139′	MJ-168	Kenturky Lake KY	34°49.300′	115°28,251′
MQ-35	Bloomington IL	34°43.963′	115°05,137′	MJ-169	Kenturky Lake KY	34°49.303′	115°28,254′
MQ-36	Bloomington IL	34°43.958′	115°05,145′	MJ-170	Wicklife KY	34°49.296′	115°28,256′
MJ-99	Toe Exit IL	34°49.287′	115°28,289′	MJ-171	Wicklife KY	34°49.299′	115°28,260′
MJ-100	Toe Exit IL	34°49.283′	115°28,278′	MJ-172	Wicklife KY	34°49.294′	115°28,262′
MJ-101	Toe Exit IL	34°49.285′	115°28,278′	MJ-173	Wicklife KY	34°49.292′	115°28,263′
MJ-102	Toe Exit IL	34°49.285′	115°28,275′	MJ-174	Wicklife KY	34°49.290′	115°28,271′
MJ-103	Toe Exit IL	34°49.287′	115°28,264′	MQ-162	Wicklife KY	34°43.970′	115°05,166′
MJ-104	Toe Exit IL	34°49.288′	115°28,263′	MQ-163	Wicklife KY	34°43.971′	115°05,167′
MQ-161	Wicklife KY	34°43.993′	115°05,192′	MQ-96	Upper Elkton RD	34°43.971′	115°05,160′

MQ-160	Wicklife KY	34°43.983′	115°05,168′	MQ-95	Upper Elkton RD	34°43.968′	115°05,156′
MQ-159	Wicklife KY	34°43.988′	115°05,170′	MQ-93	Upper Elkton RD	34°43.963′	115°05,155′
MJ-154	Knoxville TN	34°49.312′	115°28,186′	MQ-92	Upper Elkton RD	34°43.963′	115°05,148′
MJ-155	Knoxville TN	34°49.307′	115°28,197′	MQ-129	Upper Elkton RD	34°43.962′	115°05,158′
MJ-156	Knoxville TN	34°49.305′	115°28,198′	MJ-126	Ms/ALBorder	34°49.309′	115°28,161′
MJ-157	Knoxville TN	34°49.305′	115°28,203′	MJ-127	Ms/ALBorder	34°49.310′	115°28,158′
MJ-158	Knoxville TN	34°49.306′	115°28,207′	MJ-128	Ms/ALBorder	34°49.312′	115°28,150′
MJ-159	Knoxville TN	34°49.305′	115°28,212′	MJ-129	Ms/ALBorder	34°49.310′	115°28,152′
MJ-160	Knoxville TN	34°49.302′	115°28,221′	MJ-130	Ms/ALBorder	34°49.314′	115°28,141′
MQ-135	Knoxville TN	34°43.982′	115°05,167′	MJ-131	Ms/ALBorder	34°49.315′	115°28,137′
MQ-136	Knoxville TN	34°43.986′	115°05,166′	MQ-94	Ms/ALBorder	34°43.962′	115°05,154′
MQ-140	Knoxville TN	34°44.002′	115°05,170′	MQ-128	Ms/ALBorder	34°43.968′	115°05,156′
MQ-139	Knoxville TN	34°43.995′	115°05,169′	MQ-127	Ms/ALBorder	34°43.973′	115°05,160′
MQ-138	Knoxville TN	34°43.994′	115°05,167’	MQ-126	Ms/ALBorder	34°43.975′	115°05,165′
MQ-137	Knoxville TN	34°43.988′	115°05,167′	MQ-199	Ms/ALBorder	34°44.012′	115°05,217′
MJ-179	Waverlg TN	34°49.288′	115°28,300′	MQ-125	Ms/ALBorder	34°43.983′	115°05,165′
MJ-180	Waverlg TN	34°49.286′	115°28,304′	MQ-124	Ms/ALBorder	34°43.987′	115°05,168′
MJ-181	Waverlg TN	34°49.283′	115°28,307′	MJ-39	Brulington IA	34°49.300′	115°28,158′
MJ-182	Waverlg TN	34°49.286′	115°28,310′	MJ-40	Brulington IA	34°49.298′	115°28,170′
MJ-183	Waverlg TN	34°49.286′	115°28,314′	MJ-41	Brulington IA	34°49.300′	115°28,168′
MJ-184	Waverlg TN	34°49.287′	115°28,314′	MJ-42	Brulington IA	34°49.302′	115°28,166′
MJ-185	Waverlg TN	34°49.290′	115°28,309′	MJ-43	Brulington IA	34°49.306′	115°28,160′
MQ-154	Waverlg TN	34°44.011′	115°05,173′	MQ-37	Brulington IA	34°43.955′	115°05,144′
MQ-153	Waverlg TN	34°44.014′	115°05,168′	MQ-38	Brulington IA	34°43.960′	115°05,145′
MQ-152	Waverlg TN	34°44.018′	115°05,171′	MQ-185	Brulington IA	34°43.977′	115°05,174′
MQ-151	Waverlg TN	34°44.021′	115°05,174′	MQ-39	Brulington IA	34°43.967′	115°05,141′
MQ-150	Waverlg TN	34°44.028′	115°05,174′	MQ-40	Brulington IA	34°43.976′	115°05,141′
MQ-149	Waverlg TN	34°44.033′	115°05,173′	MJ-44	Hannidal MO	34°49.306′	115°28,152′
MQ-148	Waverlg TN	34°44.040′	115°05,178′	MJ-45	Hannidal MO	34°49.307′	115°28,149′
MJ-175	Sardis MS	34°49.290′	115°28,278′	MJ-46	Hannidal MO	34°49.309′	115°28,141′
MJ-176	Sardis MS	34°49.288′	115°28,286′	MJ-47	Hannidal MO	34°49.310′	115°28,137′
MJ-177	Sardis MS	34°49.288′	115°28,287′	MJ-48	Hannidal MO	34°49.309′	115°28,135′
MJ-178	Sardis MS	34°49.286′	115°28,290′	MQ-41	Hannidal MO	34°43.977′	115°05,148′
MQ-158	Sardis MS	34°43.990′	115°05,170′	MQ-42	Hannidal MO	34°43.981′	115°05,148′
MQ-157	Sardis MS	34°43.997′	115°05,172′	MQ-43	Hannidal MO	34°43.985′	115°05,147′
MQ-156	Sardis MS	34°43.998′	115°05,170′	MJ-92	ST James MO	34°49.280′	115°28,308′
MQ-155	Sardis MS	34°44.002′	115°05,170′	MJ-93	ST James MO	34°49.274′	115°28,313′
MJ-120	Upper Elkton RD	34°49.305′	115°28,205′	MJ-94	ST James MO	34°49.280′	115°28,310′
MJ-121	Upper Elkton RD	34°49.305′	115°28,200′	MJ-95	ST James MO	34°49.280′	115°28,310′
MJ-122	Upper Elkton RD	34°49.306′	115°28,193′	MJ-96	ST James MO	34°49.282′	115°28,305′
MJ-123	Upper Elkton RD	34°49.308′	115°28,183′	MJ-97	ST James MO	34°49.283′	115°28,297′
MJ-124	Upper Elkton RD	34°49.306′	115°28,180′	MJ-98	ST James MO	34°49.286′	115°28,293′
MJ-125	Upper Elkton RD	34°49.309′	115°28,175′	MQ-82	ST James MO	34°43.997′	115°05,154′
MQ-81	ST James MO	34°44.004′	115°05,156′	MJ-109	Pryor OK	34°49.301′	115°28,243′

MQ-80	ST James MO	34°44.008′	115°05,158′	MJ-110	Pryor OK	34°49.303′	115°28,241′
MQ-79	ST James MO	34°44.012′	115°05,160′	MJ-111	Pryor OK	34°49.298′	115°28,230′
MQ-78	ST James MO	34°44.018′	115°05,161′	MJ-112	Pryor OK	34°49.300′	115°28,229′
MQ-77	ST James MO	34°44.022′	115°05,159′	MQ-105	Pryor OK	34°44.020′	115°05,166′
MQ-76	ST James MO	34°44.029′	115°05,161′	MQ-104	Pryor OK	34°44.017′	115°05,164′
MJ-105	Riverton KS	34°49.293′	115°28,256′	MJ-132	Colt AR	34°49.317′	115°28,134′
MJ-106	Riverton KS	34°49.292′	115°28,254′	MJ-133	Colt AR	34°49.316′	115°28,130′
MQ-107	Riverton KS	34°44.028′	115°05,164′	MJ-134	Colt AR	34°49.318′	115°28,122′
MQ-106	Riverton KS	34°44.022′	115°05,167′	MQ-123	Colt AR	34°43.989′	115°05,769′
MJ-107	Pryor OK	34°49.296′	115°28,251′	MQ-122	Colt AR	34°43.994′	115°05,170′
MJ-108	Pryor OK	34°49.299′	115°28,244′				

Table S8. SSR screening primers used in this study

Locus		Primer Sequence (5'-3')	Type of primers	Repeat Motif	Size (bp)	GenBank Accession No.
Rp-01	F	TGCAGAAAGAGAAAGCAGAGG	EST-SSR	(TGTGAA)4	140	—
	R	CCGAACCCTTTCTGGTTAGTC				
Rp-02	F	GCTGCGTTTAATTTTGTCAGG	EST-SSR	(GAAT)4	170	—
	R	TCAATCCATCAAAGAGGAAACA				
Rp-03	F	GTGAGAAGTGGTTAGGGTTTT	EST-SSR	(CTC)7	186	—
	R	TCAAGATCACCAACGTACAA				
Rp-04	F	CTCGTGATGATGGTGTTGATG	EST-SSR	(AATGGT)4	146	—
	R	AATGGTCCAAACAACACGAAG				
Rp-05	F	CCTTGCACATTTATCCCAGAA	EST-SSR	(TCTGGC)3	158	—
	R	CGACCTCGATCTTTTCTTGTG				
Rp-06	F	TGGACAAAACATCATCGTGTG	EST-SSR	(TGAGTT)4	147	—
	R	CTCTCTTCTTTCTGCCCCTCA				
Rp-07	F	TTTTTCTCCCAACGAAACAAA	EST-SSR	(CT)10	144	—
	R	TGATGTGTTGTACGGAGGTGA				
Rp-08	F	TCAGGTGCATAAGCTCATTACTTC	EST-SSR	(AAAAT)4	152	—
	R	GGTTGTCAGATGAAATGCACA				
Rp-09	F	CGTTTAGAAGCTGAGGCAGAA	EST-SSR	(CTTT)5	153	—
	R	TGAGATATCTTAGTGCAGGAGCA				
Rp-10	F	GGCATGTGGCTATGAAGATGT	EST-SSR	(CCTTT)4	154	—
	R	TCAGTGGGACTTGGTTTCTTG				
Rp-11	F	GAAGCTATCACCGCAAATGAA	EST-SSR	(AG)10	150	—
	R	GTCGAAGTGCGTCCTAGATCA				
Rp-12	F	AAGAGTCATCACGGAGACCAA	EST-SSR	(AGCAGA)4	150	—
	R	GGAGTCCAATTAAGTGCGAGA				

Rp-13	F	CATTTCCGATTTCCAATTCCT	EST-SSR	(CTCTTC)4	151	—
	R	GCCGAGGACTCGGTAGAAGT				
Rp-14	F	TTAGCACGAACCTGGTTATGG	EST-SSR	(TGCAAC)4	151	—
	R	CACTTCATTTGGTTCCTTGAGA				
Rp-15	F	TTAACTAATGCGGCGAGAAGA	EST-SSR	(TCAC)5	119	—
	R	GAGAGGAAGTGTGCGAAACAA				
Rp-16	F	TATGAGACAGTGTTGGTTGGT	EST-SSR	(TTCAGT)4	175	—
	R	CGTGCCAGAAGAGTATAACAG				
Rp-17	F	GTAAGTCTGCAAAGAAGACCA	EST-SSR	(AACCA)4	150	—
	R	GCTTTTCACCTATCAACTCAA				
Rp-18	F	GGATGAACTTTGGCAATCCTT	EST-SSR	(GGTCAG)4	158	—
	R	AATTTGTTGGGAATGCTGTTG				
Rp-19	F	CAGGAGTGGCAGCATTAGTGT	EST-SSR	(AGGCTG)4	123	—
	R	CACAACAAGCACATTTTGCAC				
Rp-20	F	TTTCTTGGCTTGCTTTTGCTA	EST-SSR	(GCAGCT)3	145	—
	R	TCTTGGATACGCAAGGTTGTC				
Rp-21	F	TATGATCACGTCCCCTAATGC	EST-SSR	(CCA)7	146	—
	R	AAGTGGAAAGAAATGGGATGG				
Rp-22	F	GGTAAGGTGAAGGAGGTGGAG	EST-SSR	(AGGGTT)4	150	—
	R	AGCTTGGTCTCCTAGGTCGTC				
Rp-23	F	GGAGGAGCAACCATCTGTGTA	EST-SSR	(AGAAGT)4	146	—
	R	CTCCCTCTTCATCCTCACCTC				
Rp-24	F	TGCACATATTTGCCTGGTTTA	EST-SSR	(AATA)4	160	—
	R	AAAATGAGCATGACACAACCA				
Rp-25	F	CGGCAACAAGTTGAGAAGAAC	EST-SSR	(AAAG)5	139	—
	R	GGCTCACAAACCAACCTATGA				

Rp-26	F	GCTGCAAGCAAAGGATCTTAC	EST-SSR	(ATGATA)4	139	—
	R	CCTCATCATCCTCGTCATCAT				
Rp-27	F	TGGACAAAACATCATCGTGTG	EST-SSR	(TGAGTT)4	147	—
	R	CTCTCTTCTTTCTGCCCCTCA				
Rp-28	F	CTTGGTCTAGAAAGTCCTGCT	EST-SSR	(CAG)7	151	—
	R	GGTCATCAAGGTTAGTTGGAT				
Rp-29	F	CCTGATGATCAAAACGACGAC	EST-SSR	(GATC)4	148	—
	R	GGAGGTGACCCCTCTTATCCT				
Rp-30	F	TTGAACCAAAACTGGAAGAGC	EST-SSR	(GCT)8	151	—
	R	GCACCGTACAGTTACCCTATCC				
Rp-31	F	GACCCCATTTTTCTCAAGGAC	EST-SSR	(ATT)7	140	—
	R	TTGGATAAGTCGGTGAAGGTG				
Rp-32	F	CCACGTGGTTCTTCAAACATT	EST-SSR	(GTG)7	163	—
	R	CAACAACAACCCACAAACACA				
Rp-33	F	CAAACAGTCTCATGGAAATGGA	EST-SSR	(ATC)7	141	—
	R	GGGTTGGTATTGTTGGGAAAT				
Rp-34	F	AGGATATTAGCCAAGTCCATC	EST-SSR	(TGGTGA)4	164	—
	R	AGTAACCATCACCACAATCAC				
Rp-35	F	TCAGACGTGGTAGAGCAGTGTT	EST-SSR	(CACAC)4	152	—
	R	ATTTGTTTTTGGGGGAGATTG				
Rp-36	F	CGTTTCAGCCATTGATTTTGT	EST-SSR	(GAATC)5	141	—
	R	GATCATCACCGTCCACCTTC				
Rp-37	F	TGTCGTCATTTTATTTTACCC	EST-SSR	(GAACGA)4	152	—
	R	CTCACCCTTTTTATTTCCATT				
Rp-38	F	TCCATTCCCTGGTTTCTTCTT	EST-SSR	(TC)10	150	—
	R	AGCACAATTTCCTCAGTGCAG				

Rp-39	F	TTAAAGAATGTTCCGTTCAGA	EST-SSR	(AAGAGG)3	152	—
	R	GAGAAGATAGCCTCCTAGCTG				
Rp-40	F	TCATTGGACATCCCTCCATAA	EST-SSR	(TAA)8	139	—
	R	GGCTCGACATGGTTGATTTT				
Rp-41	F	AACTCACCCAATTGCACACTC	EST-SSR	(CCA)7	143	—
	R	GAGCAAGAGCTAAAGCAGCAA				
Rp-42	F	CTTCGCAATCCTCACTCTTTG	EST-SSR	(AATC)4	169	—
	R	CTTACCCAGAAGCCAACAATG				
Rp-43	F	CAAAGCAGAGAGAATGTATGG	EST-SSR	(CAAAAT)4	155	—
	R	ATCCCTTGCTCCTTGTAATAG				
Rp-44	F	TATCTGGGAGAATCGAGAGCA	EST-SSR	(ATCA)5	145	—
	R	CCACCATGGTTGTCCTTCTAA				
Rp-45	F	GGGTTGAGGAAGAGAGGAGAA	EST-SSR	(TTC)7	156	—
	R	AAAAATCGAATCGTGTTGGTG				
Rply1	F	AGTTCGCAAAGGAAGGAG	EST-SSR	(AAG)6	243	—
	R	GGTAACAAGCACCAGCAA				
Rply2	F	TGTGAATGGTTGGTGGACAT	EST-SSR	(CCA)6	161	—
	R	CGTTGCTTGGAGGAGAATAA				
Rply3	F	GCCTCATAAATAAAAGGAACG	EST-SSR	(GTGGT)4	246	—
	R	CTGCCATTGGTAACTGGTAAA				
Rply5	F	GAGTCATGCCCTTTGTATGTT	EST-SSR	(ATG)8	242	—
	R	TGTCACCTTCAAGTCCCTATT				
Rply8	F	TCCCTACATAAAACTCCAAA	EST-SSR	(CT)14	120	—
	R	TCATTAAGTCAGCACTCACAG				
Rply11	F	ATAGCAGTGGGTGAACAAG	EST-SSR	(AG)10	157	—
	R	GAAACCGAAGCTCAAGTAG				

Rply15	F	GGCACAAGAACAGAACAAA	EST-SSR	(CAT)7	239	—
	R	GTGGTGGATAAGGATAAGC				
Rply16	F	CGTCGGAGTGCTGTTATG	EST-SSR	(GAA)7	242	—
	R	AGCTTATGGCAAAGAGGG				
Rply17	F	TCTGATCCTGATGGTGCTG	EST-SSR	(GAT)6	255	—
	R	GTCGTCGTGTCCTTCTGTC				
Rply18	F	GCTCGTCAATATGGCAAAC	EST-SSR	(TGCAG)4	178	—
	R	CAACATTATCACCCAACCC				
Rply21	F	TCAGTTGAACCAGCAGAGT	EST-SSR	(TGTA)5	187	—
	R	ATATCGCCATGTAAAGCAG				
Rply22	F	ATCACCATCTGTTCCTCCAC	EST-SSR	(ACCTG)4	120	—
	R	TTCTCCTCAGCCACTTCTTT				
Rply27	F	CATTTAGTTGGAGGCATTG	EST-SSR	(TAT)6	152	—
	R	AAGATGGAAGTGACGAGGA				
Rply28	F	TGGAGGGTTAGATTTGGATGT	EST-SSR	(TGAGC)4	150	—
	R	AGGATGGAGAGTAAAGGGTTG				
Rply31	F	CATTCTTCCCAATCTCCTTT	EST-SSR	(TCC)5	157	—
	R	GTTTCATTTTCGCATTCTGT				
Rply32	F	TGGGATCTTTGGAGGTAAT	EST-SSR	(GTT)5	239	—
	R	TAGAGGCTCAATGGTTTCA				
Rply33	F	GCCCATTCTGTTGGTTCT	EST-SSR	(ACT)7	266	—
	R	GGTTATGTGCCCTCGTGT				
Rply36	F	CGTTGAAAGAGGGAAAGCAG	EST-SSR	(CT)7	281	—
	R	GAAATCGACGCCAATGGTAC				
Rply43	F	GTTTACGGCGTTGTTGAGTT	EST-SSR	(AT)6	147	—
	R	CTAATTGCTTGCATTTCCCT				

Rply44	F	AAAGTGAGACATCCAAGTTC	EST-SSR	(CT)7	153	—
	R	CAACCTTCCATTGTTTACAC				
Rply45	F	CAACTCGTTTTCCTTCTTCA	EST-SSR	(TA)8	177	—
	R	ACCGAATCAGATCACATCAA				
Rply49	F	CCCCGTACAGTTCCATCT	EST-SSR	(CT)8	120	—
	R	GACCTCGTAAAAGCCACC				
Rply50	F	GCGAAAACGACAAGGAGATA	EST-SSR	(TC)8	290	—
	R	TTTGAGGAGCAACAAAGAGC				
Rply53	F	TGTTCTCCTCACCTTCCCTT	EST-SSR	(GA)6	250	—
	R	GTTCATGGCTCTTGTCTTCC				
Rply60	F	ATCTTTGACGCTTCTCGC	EST-SSR	(TC)6	135	—
	R	CATTTCTTCTTCAGCCTGTG				
RP035	F	M13-GGAGTGGAATGCATGCTCTCATG	G-SSR	(TC)15	89–112	AB353927
	R	TCCAAATGGAAACTCCCTTGAAACAGC				
RP102*	F	M13-CCAAATCTCAAAATGTGCTAAGTAGC	G-SSR	(GA)12	205–211	AB353928
	R	ACTTGGGCTATGGTATTGCA				
RP106	F	M13-AAACTGAATTATATCCCTTTACGGC	G-SSR	(GT)9	143–154	AB353929
	R	GCATATATCCACCAGATACCCG				
RP109	F	M13-GAGGAATCACAAAACCGTTTGG	G-SSR	(AG)17	136–160	AB353930
	R	TGGGATTTGAGAGAGTGGTGGTG				
RP150	F	M13-TCGTTGGATCAACATGCATGG	G-SSR	(TC)3TT(TC)12	199–217	AB353931
	R	ACAGAACCCTAACCCTAGCA				
RP206	F	M13-GCCAAATCCCATTAGATCACAGTTGA	G-SSR	(GT)9	222–246	AB353932
	R	AGAAGTTAGACTTACGTGCTGC				
RP200	F	M13-GGTTTCTTTGTTCACCTGCTCTGG	G-SSR	(AG)23	160–198	AB353933
	R	ACCTACGTGTCCACGGCTCT				

RP032	F	M13-GCATATTGCATATGCGCTTGTG	G-SSR	(TG)13	109–135	AB353934
	R	TCCCTGAAGCTCATAACTGTCATGTG				
RP211*	F	M13-TGTAATCCATGTAGTTGACCCCAC	G-SSR	(TC)7A(AC)8	196–201	AB353935
	R	TGATTACTCTGCATGCATGTG				
RP165*	F	M13-TTAGATGTTGCAAGTGCTGAGG	G-SSR	(TG)8	146–159	AB353936
	R	ACAATGCCTCAATGCAGC				
RP01B	F	M13-ACCAATTAGGTAACGTCAGC	G-SSR	(CT)16	172–192	AB353937
	R	TGTTCACTGACAAAGCTG				
Rops02	F	CAGAACTGTGGAGAATAATTCTGAACCG	G-SSR	(AC)13(AT)4	107–138	AB075029
	R	CGCCATCTGTTAGTTTGTTGC				
Rops04*	F	GTCTAATTTCACTTTTCTCACGAG	G-SSR	(AC)10	105–110	AB075030
	R	GGACACCACCRAAATTCTACC				
Rops05	F	TGGTGATTAAGTCGCAAGGTG	G-SSR	(AC)2GC(AC)7	120–138	AB075031
	R	GTTGTGACTTGTACGTAAGTC				
Rops06	F	CTAAGGAGGTGCTGACCCTC	G-SSR	(GT)3ACA(GT)11	117– 144	AB075032
	R	TTAATCTGTGATGGGACACTG				
Rops08	F	TTCTGAGGAAGGGTTCCGTGG	G-SSR	(CA)8TA(CA)3	191–205	AB075033
	R	GTTAAAGCAACAGGCACATGG				
Rops09*	F	CTCCAGGTCACTCGATTGAGG	G-SSR	(TA)6A4(TA)2(TG)	89–150	AB075034
	R	TTTCTCATTTGATACGACCCC				
Rops10	F	AACTTTTTCCGTATAGGGGTC	G-SSR	T12AAT4	182–187	AB075035
	R	GAGTTTTACACTTGGTCAAACC				
Rops15	F	GCCCATTTTCAAGAATCCATATATTGG	G-SSR	(CT)20	112–254	AB120731
	R	TCATCCTTGTTTTGGACAATC				
Rops16	F	AACCCTAAAAGCCTCGTTATC	G-SSR	(CT)13	195–223	AB120732
	R	TGGCATTTTTTGGAAGACACC				

Rops18	F	AGATAAGATCAAGTGCAAGAGTGTAAG	G-SSR	(AC)8	135–219	AB120733
	R	TAATCCTCGAGGGAACAATAC				

Table S9. *Robinia pseudoacacia* L. samples analyzed using SSR primer pairs

No.	Varieties	Sample collection sites
1	*Robinia hispida* L.	Shandong
2	49	Shandong
3	285	Shandong
4	290	Shandong
5	158	Mengjin
6	232	Mengjin
7	Q28	Mengjin
8	YC9	Mengjin
